# Identification of necroptosis-related subtypes, development of a novel signature, and characterization of immune infiltration in colorectal cancer

**DOI:** 10.3389/fimmu.2022.999084

**Published:** 2022-12-05

**Authors:** Mengyu Sun, Xiaoyu Ji, Meng Xie, Xiaoping Chen, Bixiang Zhang, Xiangyuan Luo, Yangyang Feng, Danfei Liu, Yijun Wang, Yiwei Li, Bifeng Liu, Limin Xia, Wenjie Huang

**Affiliations:** ^1^ Department of Gastroenterology, Hubei Key Laboratory of Hepato-Pancreato-Biliary Diseases, Institute of Liver and Gastrointestinal Diseases, Tongji Hospital of Tongji Medical College, Huazhong University of Science and Technology, Wuhan, Hubei, China; ^2^ Hubei Key Laboratory of Hepato-Pancreato-Biliary Diseases, Hepatic Surgery Center, Tongji Hospital, Tongji Medical College, Huazhong University of Science and Technology, Clinical Medicine Research Center for Hepatic Surgery of Hubei Province, Wuhan, Hubei, China; ^3^ Key Laboratory of Organ Transplantation, Ministry of Education and Ministry of Public Health, Wuhan, Hubei, China; ^4^ The Key Laboratory for Biomedical Photonics of MOE at Wuhan National Laboratory for Optoelectronics-Hubei Bioinformatics and Molecular Imaging Key Laboratory, Systems Biology Theme, Department of Biomedical Engineering, College of Life Science and Technology, Huazhong University of Science and Technology, Wuhan, China

**Keywords:** colorectal cancer, necroptosis, signature, subtypes, immune infiltration

## Abstract

**Introduction:**

Necroptosis, a type of programmed cell death, has recently been extensively studied as an important pathway regulating tumor development, metastasis, and immunity. However, the expression patterns of necroptosis-related genes (NRGs) in colorectal cancer (CRC) and their potential roles in the tumor microenvironment (TME) have not been elucidated.

**Methods:**

We explored the expression patterns of NRGs in 1247 colorectal cancer samples from genetics and transcriptional perspective. Based on a consensus clustering algorithm, we identified NRG molecular subtypes and gene subtypes, respectively. Furthermore, we constructed a necroptosis-related signature for predicting overall survival time and verified the predictive ability of the model. Using the ESTIMATE, CIBERSORT, and ssGSEA algorithms, we assessed the association between the above subtypes, scores and immune infiltration.

**Results:**

Most NRGs were differentially expressed between CRC tissues and normal tissues. We found that distinct subtypes exhibited different NRGs expression, patients’ prognosis, immune checkpoint gene expression, and immune infiltration characteristics. The scores calculated from the necroptosis-related signature can be used to classify patients into high-risk and low-risk groups, with the high-risk group corresponding to reduced immune cell infiltration and immune function, and a greater risk of immune dysfunction and immune escape.

**Discussion:**

Our comprehensive analysis of NRGs in CRC demonstrated their potential role in clinicopathological features, prognosis, and immune infiltration in the TME. These findings help us deepen our understanding of NRGs and the tumor microenvironment landscape, and lay a foundation for effectively assessing patient outcomes and promoting more effective immunotherapy.

## Introduction

Colorectal cancer is a malignant tumor that poses a great risk to human health and ranks in the top three of all cancer types globally in terms of incidence and lethality (1). The progression from normal mucosa to adenomatous polyps to colorectal cancer is a step-by-step process characterized by the accumulation of genetic mutations. Genetic and epigenetic changes that disrupt the balance between cell proliferation and cell death play an extremely important role in the events that drive tumor phenotypes (2).

Cell death is a fundamental physiological process in all living organisms. It plays an integral role in embryonic development, organ maintenance, aging, and extending to the coordination of immune responses and autoimmunity (3). As a type of programmed cell death similar to ferroptosis or pyroptosis, necroptosis has recently become an important pathway that has been extensively studied in regulating tumorigenesis and progression. The necroptosis signaling pathway plays a role in multiple important events, such as tumor development, immune response, necrosis, and metastasis (4, 5). However, necroptosis may exert anti-tumor or pro-tumor effects, depending on the tumor type. Necroptosis is a caspase-independent form of programmed death that is triggered by multiple stimuli and different pathways (6). RIPK1, RIPK3, and MLKL are essential components of the necroptotic process, leading to membrane leakage and cytokine release (7).

Surgery and chemotherapy are the most common treatment modalities for colorectal cancer. When pro-apoptotic chemotherapy fails due to the development of drug resistance, necroptosis-based tumor therapy may become a new alternative therapy (8). An important reason for the development of intrinsic and acquired chemoresistance is the destruction of the apoptosis mechanism caused by caspase inhibition and deficiency (9). Therefore, necroptosis is a promising tool capable of killing cancer cells with defective and inhibited caspase. Cells undergoing necroptosis are also involved in the activation of the immune system, particularly antigen presentation and cross-priming of CD8^+^ T cells (10, 11). *In vivo* and *in vitro* experiments also showed that necrotic tumor cells induce antitumor immunogenicity through cross-priming and proliferation of CD8^+^ T cells (12). This suggests that there is also a close link between necroptosis and tumor immunity.

Numerous necroptosis inducers have been identified, laying the foundation for studying new modes of tumor death and providing new therapeutic approaches. However, most of these studies were based on *in vitro* experiments. There is still a lack of *in vivo* necroptosis markers for research, and there is a great need to further identify novel necroptosis-related biomarkers and study their effects on tumor cells and the microenvironment. At present, studies have built models based on necroptosis-related genes in colorectal cancer and demonstrated good predictive effects (13–16). This study focused on necroptosis-related messenger RNAs that play important roles in colorectal cancer. This study comprehensively evaluated RNA sequencing data of necroptosis-related genes, and mainly used three algorithms (ssGSEA, CIBERSORT, and ESTIMATE) to obtain a comprehensive overview of the immune landscape of CRC. We first performed unsupervised clustering of the samples based on the expression levels of 67 NRGs to classify patients into two NRG-related subtypes. Next, the samples were divided into two gene subtypes by consensus clustering based on the differentially expressed genes identified between two NRG-related subtypes. We further constructed a necroptosis-related signature to assess the prognostic risk and immune status of patients, thereby enabling individualized prediction of patients’ prognosis and responsiveness to immunotherapy. With the in-depth study of the mechanism of necroptosis in tumors, targeting strategies based on necroptosis are highly promising to be another effective tool for cancer treatment.

## Methods

### Data collection and pre-processing

Gene expression and clinically relevant information were obtained from the Cancer Genome Atlas (TCGA) and Gene-Expression Omnibus (GEO) databases. Three independent CRC cohorts (TCGA-COAD, GSE17538, and GSE39582) were collected for this study. We acquired gene expression data in FPKM (Fragments per Kilobase Million) format from TCGA and converted them to TPM (Transcripts per million) format, downloaded normalized matrix files from the GEO database, and removed batch effects between these datasets using the R package SVA. We downloaded clinical information of the three datasets, and detailed clinical information was provided in **Supplementary Tables 1-3**. We extracted survival status and survival time from clinical information, and excluded data with a follow-up time fewer than 31 days and duplicate data. Additionally, we downloaded the tumor immune subtype and stemness score files from the UCSC database for subsequent analysis.

### Expression and prognosis of necroptosis-related genes (NRGs) in CRC

By searching necroptosis-related literature, we identified 67 necroptosis-related genes (17). To investigate whether NRGs play a role in the development of CRC, we compared NRGs expression in normal and CRC tissues, and we subsequently performed Kaplan-Meier survival analysis and univariate COX analysis to determine the prognostic value of NRGs.

### Consensus clustering analysis of NRGs

Unsupervised clustering analysis was performed to identify different NRG-related molecular subtypes based on the gene expression profiles of 67 NRGs, and patients were classified for follow-up studies. We used a consensus clustering algorithm to determine the number of clusters (K) and their stability, using the K value corresponding to the flattest cumulative distribution function (CDF) curve as the determined number of clusters. The R package consusclusterplus was utilized to perform the above analysis steps. Principal component analysis was used to determine whether there was good discrimination between subtypes. To investigate the biological process differences among NRG-related molecular subtypes, we performed gene set variation analysis (GSVA) based on gene set c2.cp.kegg.v7.4.symbols.

### Identification of differentially expressed genes (DEGs) between NRG subtypes

The above consensus clustering analysis divided patients into different NRG-related molecular subtypes and DEGs between NRG subtypes were identified using the R package limma with screening criteria of fold-change ≥ 1.5 and adjusted P-value < 0.05.

### Unsupervised clustering analysis of gene subtypes

To explore the biological functions of differentially expressed genes between NRG-related molecular subtypes, we performed functional enrichment analysis using the clusterProfiler package, including GO (Gene Ontology) and KEGG (Kyoto Encyclopedia of Genes and Genomes) analysis. The screening criteria were P < 0.05 and FDR < 0.05. Univariate Cox regression analysis was performed on DEGs, and genes associated with CRC prognosis were selected for unsupervised clustering of samples to identify gene subtypes. The number and stability of gene clusters were determined by the consensus clustering algorithm.

### Evaluation of immune infiltrating cells in TME of CRC

ssGSEA (single sample GSEA) is an implementation method mainly proposed for a single sample that cannot do Gene Set Enrichment Analysis (GSEA). We used ssGSEA to explore the infiltration of various types of immune cells and immune-related functions in CRC samples. The R packages limma, GSEABase and GSVA were used to perform the above analysis. CIBERSORT is a deconvolution algorithm. The R package CIBERSORT can calculate the proportion of different types of immune cells in a sample based on the Leukocyte signature matrix (LM22) containing 547 reference genes. The ESTIMATE algorithm infers tumor purity and cell density from the RNA sequencing data of the samples. Based on the R package ESTIMATE, we assessed the immune and stromal content (immune and stromal scores) in each sample. In addition, we also collected 47 immune checkpoint genes to compare their expression in different subtypes.

### Construction of the necroptosis-related signature (NR-signature)

We first randomly divided all samples into two groups: the training group (n=570) and the testing group (n=570). The training group was used to learn the sample characteristics and construct the signature, and the testing group was used as a validation cohort to test the prediction performance of the signature.

Based on the DEGs screened between different NRG subtypes, we performed a univariate Cox analysis to further identify the DEGs associated with CRC prognosis. Next, we performed a 10-fold cross-validation lasso (least absolute shrinkage and selection operator) regression and Cox proportional hazards regression analysis. Lasso regression was run for 1000 cycles to obtain the gene combination with the smallest cross-validation error, followed by Cox analysis and model construction. The final risk score was calculated as follows:


$$\text{RiskScore}=\sum_{i=1}^{N}(Exp_{i}*W_{i}),$$

where Exp is the expression value of each gene in the signature, and W is the coefficient of multiple cox regression analysis for each gene. The median risk score in the training set was used as the cut-off value, and patients were divided into a high-risk group and a low-risk group. Survival curves were plotted to evaluate the overall survival (OS) of two groups, and the predictive performance of this signature for 1-, 3-, and 5-year survival was evaluated using the receiver operating characteristic (ROC) curve. Subsequently, the predictive NR-signature was applied to the testing group and the whole samples for validation.

### Validation and comparison of NR-signature

Many prognostic models have been constructed and demonstrated good predictive performance in CRC. To investigate whether the signature based on necroptosis is effective and complementary to existing studies, we compared our model with other phenotype-based models, including those based on immune, autophagy, pyroptosis, ferroptosis, aging, and metabolism genes (14, 18–27). Based on the TCGA database, we calculated and compared the concordance index (C-index) of several signatures, and we plotted the ROC curve and survival curve of each signature to visualize the predictive effect.

### GSEA analysis, genomic mutation analysis, clinical relevance, and immune correlation analysis of NR-signature

We performed GSEA analysis on all samples to enrich the respective involved signaling pathways in high and low risk groups based on the KEGG gene set with clusterProfiler package. To explore the tumor mutational burden (TMB) in CRC, we calculated the total number of non-synonymous mutations in the samples from TCGA database. The R package maftools was used to draw the oncoprint of gene mutations in high and low risk groups. We also investigated the correlation of risk groups with clinical indicators to see if there were differences in the proportion of high- and low-risk samples in different tumor stages and immune subtypes. To explore the relationship between risk status and immune cells, we assessed the status of immune cell infiltration and immune function in samples using multiple methods including ssGSEA and the CIBERSORT algorithm. In addition, we also used various methods such as XCELL, TIMER, QUANTISEQ, MCPCOUNTER, EPIC, CIBERSORT-ABS, and CIBERSORT to analyze the correlation between risk scores and infiltrating immune cells based on TCGA samples. We also calculated the TIDE score and Dysfunction score of the TCGA samples based on the online website TIDE (http://tide.dfci.harvard.edu/) to evaluate the immune escape and immunotherapy of the samples in the high and low risk groups.

### Pan-cancer analysis of genes in NR-signature

We compared the differential expression of genes in normal and cancer tissues in pan-cancer. The correlation of gene expression with immune microenvironment scores (including immune and stromal scores, ESTIMATE score, and tumor purity), Stemness Score (including RNAss: RNA expression-based and DNAss: DNA methylation-based), and immune subtypes were calculated on a pan-cancer scale.

### Quantitative real-time PCR (RT-qPCR)

We obtained cancer and normal tissue samples from eight CRC patients who underwent curative resection at Tongji Hospital of Tongji Medical College (Wuhan, China). The MolPure^®^ Cell/Tissue Total RNA Kit (Yeasen) was used to extract total RNA, which was then reverse transcribed with the Hifair^®^ III 1st Strand cDNA Synthesis Kit (gDNA digester plus, Yeasen) in accordance with the manufacturer’s protocols. The target sequence was amplified with real-time PCR with the Hieff^®^ qPCR SYBR Green Master Mix(Low Rox Plus, Yeasen). The cycling parameters used were 95°C for 5 min, 95°C for 10 s, and 60°C for 30 s for 40 cycles. Melting curve analyses were performed, and Ct values were determined during the exponential amplification phase of real-time PCR. The 2^–ΔΔCt^ method was used to determine relative fold changes between tumor tissues and normal tissues as the following equation: 2 ^–ΔΔCt^ (ΔΔCt = ΔCt^tumor^ – ΔCt^normal^). The primer sequences were listed in **Supplementary Table 5**.

### Statistical methods

All statistical analysis and graphing were performed by R-4.1.2. Wilcoxon rank-sum test and Student’s t test were used for comparison between the two groups. Kruskal-Wallis test was used for comparison among more than two groups of samples. The Kaplan-Meier method was used to plot survival curves for prognostic analysis, and the log-rank test was used to determine the significance of differences. The correlation test was performed using Spearman correlation analysis and distance correlation analysis. Comparisons of composition ratios among groups were performed by chi-square test. All heatmaps were plotted by the R package pheatmap. All statistical P values were two-tailed, and P < 0.05 was used as the truncated value.

## Results

### Differential expression and survival analysis of NRGs

**Figure 1:**
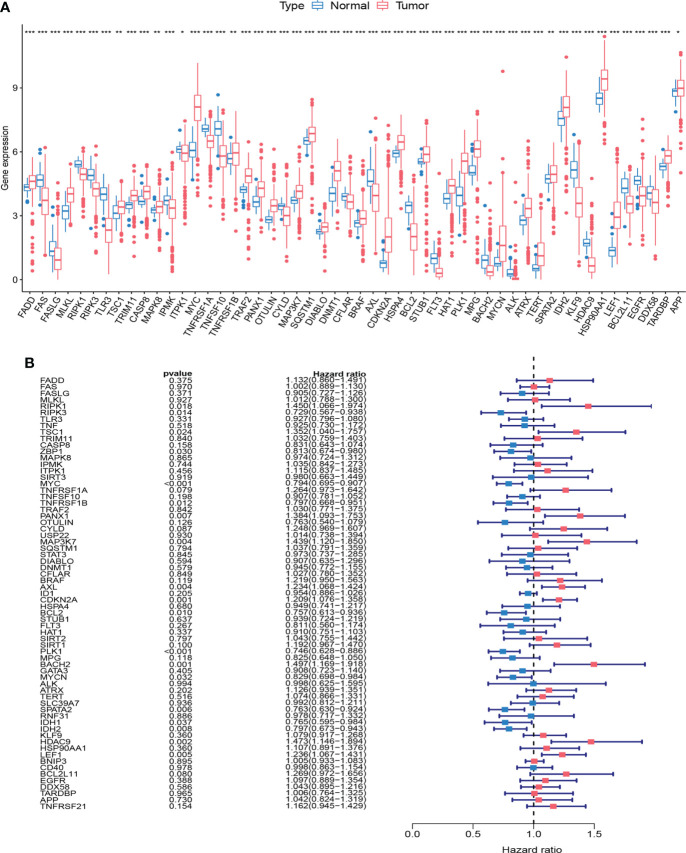
Expression and survival information of necroptosis-related genes (NRGs) in colorectal cancer. **(A)** Expression distribution of 67 NRGs in normal and cancer tissues. **(B)** Univariate COX analysis of 67 NRGs. ***P < 0.001; **P < 0.01; *P < 0.05.

We first compared the expression of 67 NRGs in 473 CRC tissues and 41 normal tissues based on the TCGA database, of which 52 genes were differentially expressed (**Figure 1A**). Among them, MLKL was highly expressed in cancer tissues, while RIPK1 and RIPK3 were lowly expressed in cancer tissues. To provide a more comprehensive landscape of the expression of NRGs, we jointly analyzed TCGA and GTEx database through GEPIA to expand the sample size. NRGs with differential expression exceeding 1.5 times were shown in **Supplementary Figure 1**. A total of 1247 tumor samples were obtained after combining TCGA and GEO data, and we further analyzed whether there was an impact of NRGs on the prognosis of CRC patients. We performed univariate Cox analysis (**Figure 1B**) and Kaplan-Meier survival analysis (**Supplementary Figure 2**), respectively (**Supplementary Table 4**). The analysis results showed that the expression of some NRGs was related to the survival of patients. The high expression of RIPK1 was associated with poor prognosis, while the high expression of RIPK3 represented a good prognosis, and the MLKL expression was not related to the survival time of patients.

### Identification of NRG subtypes in CRC

**Figure 2:**
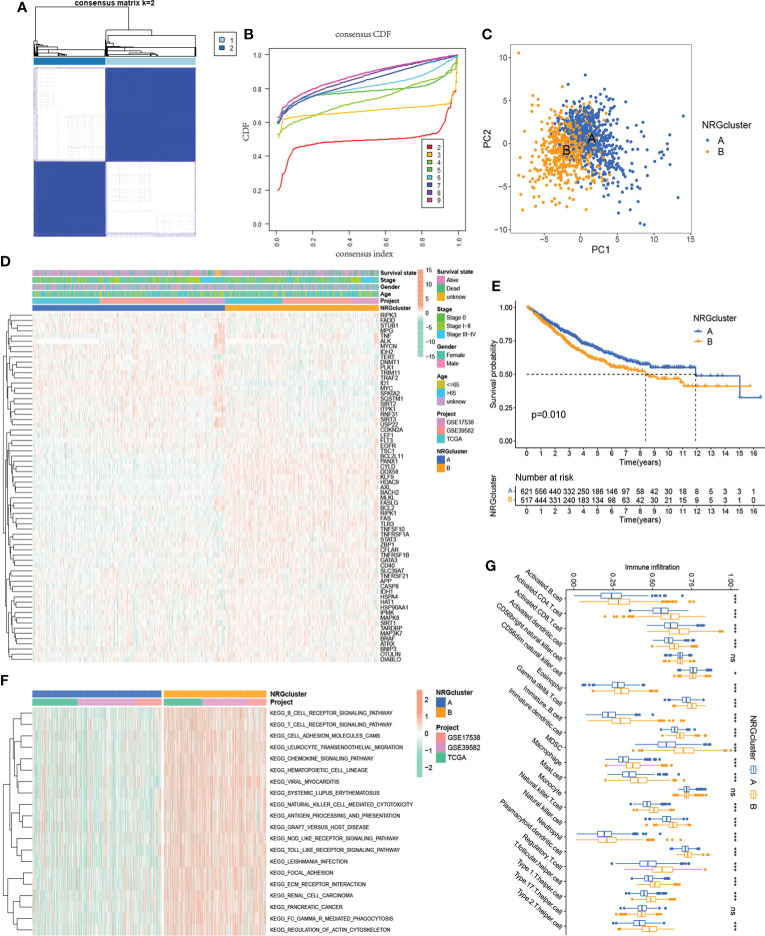
Identification of two NRG subtypes. **(A)** Consensus matrix heatmap of sample clustering under k = 2. **(B)** Cumulative distribution function **(C, D, F)** curve with the number of subtypes k = 2 to 9. **(C)** Principal component analysis showing the difference in transcriptomes between the two NRG subtypes. **(D)** Heatmap showing differences in the expression of 67 NRGs between distinct NRG clusters. **(E)** Survival analysis of patients in two NRG clusters. **(F)** GSVA enrichment analysis showing the potential biological change between distinct NRG clusters. **(G)** TME immune-infiltrating characteristics and transcriptome traits of two NRG clusters. ***P < 0.001; *P < 0.05; ns P ≥ 0.05.

To further explore the expression characteristics of NRGs in colorectal cancer, we qualitatively classified patients based on the expression profiles of 67 NRGs using the R package ConensusClusterPlus. By consensus clustering algorithm, a cluster number (K value) of 2 was the best choice to classify the whole samples into NRG cluster A (n=695) and NRG cluster B (n=552) (**Figures 2A, B**). The principal component analysis demonstrated significant differences of necroptosis gene profile between the two clusters (**Figure 2C**), and the heatmap showed the expression of NRGs in two NRG clusters (**Figure 2E**). Kaplan-Meier survival analysis indicated that patients with NRG cluster A had better overall survival (P = 0.010, **Figure 2D**). To explore the potential biological change between distinct NRG clusters, we applied GSVA enrichment analysis, which showed that NRG cluster B was significantly enriched in immune-related pathways, including B_CELL_RECEPTOR_SIGNALING_PATHWAY, T_CELL_RECEPTOR_SIGNALING_PATHWAY, LEUKOCYTE_TRANSENDOTHELIAL_MIGRATION, CHEMOKINE_SIGNALING_PATHWAY, NATURAL_KILLER_CELL_MEDIATED_CYTOTOXICITY, ANTIGEN_PROCESSING_AND_PRESENTATION, NOD_LIKE_RECEPTOR_SIGNALING_PATHWAY, TOLL_LIKE_RECEPTOR_SIGNALING_PATHWAY and REGULATION_OF_ACTIN_CYTOSKELETON (**Figure 2F**). To further investigate the role of NRGs in TME of colorectal cancer, we applied the ssGSEA algorithm to evaluate the association between NRG subtypes and immune cell subpopulations, and the results showed significant differences in the infiltration of most immune cells between different clusters (**Figure 2G**). Immune cell infiltration was more abundant in NRG cluster B, including Activated.B.cell, Activated.CD4.T.cell, Activated.CD8.T.cell, Activated.dendritic.cell, Eosinophil, Gamma.delta.T.cell, MDSC (Myeloid-derived suppressor cell), Macrophage, Monocyte, Natural.killer.T.cell, Natural.killer.cell, Neutrophil, Regulatory.T.cell (Treg). In addition, the expression of immune checkpoint genes was generally upregulated in cluster B.

### Identification of gene subtypes based on differentially expressed genes

**Figure 3:**
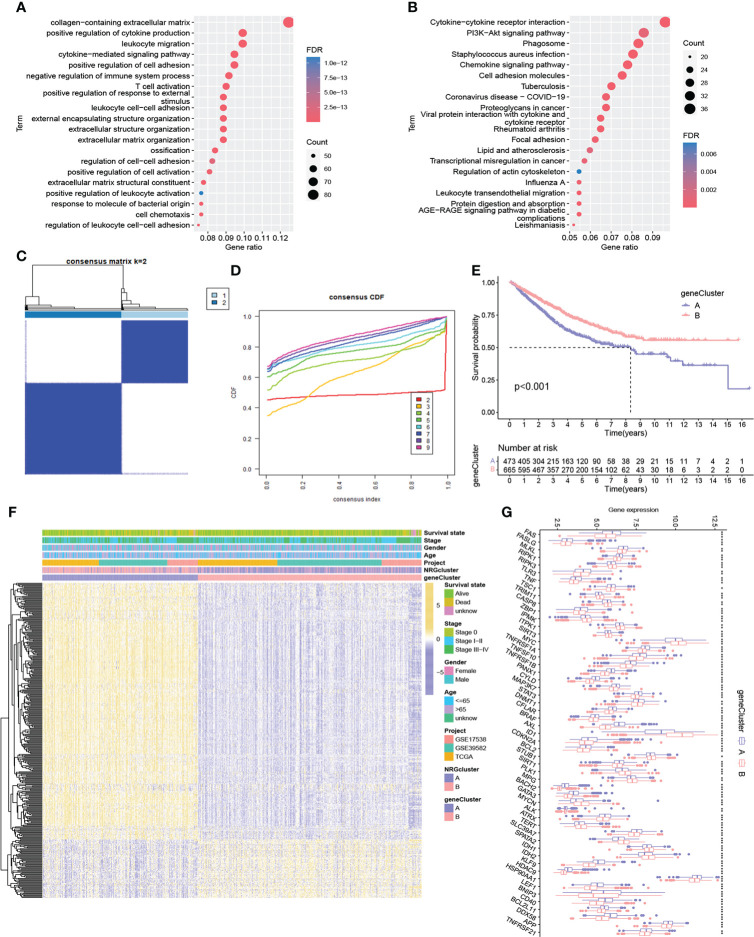
Identification of two gene subtypes. **(A-B)** GO and KEGG enrichment pathway of differentially expressed genes between two NRG subtypes. **(C)** Consensus matrix heatmap of sample clustering under k = 2. **(D)** CDF curve with the number of subtypes k = 2 to 9. **(E)** Survival analysis of patients in two gene clusters. **(F)** Heatmap showing differences in the expression of necroptosis-related signature genes between distinct gene clusters. **(G)** Differences of 67 NRG expression between two gene clusters. ***P < 0.001; **P < 0.01; *P < 0.05; ns P ≥ 0.05.

To reveal the potential biomolecular characteristics underlying different NRG subtypes, we identified 702 NRGcluster-related DEGs based on R package limma, followed by GO and KEGG functional enrichment analyses (**Figures 3A, B**). The results showed that these DEGs were mainly enriched in biological processes such as immune-related processes, cytokines-related processes, chemokines-related processes, and tumor signaling pathways, suggesting that necroptosis may play an important role in tumor development and immune regulation. We then performed a univariate Cox regression analysis to identify 361 NRGcluster-related DEGs with prognostic value, which were defined as necroptosis-related signature genes (NRSGs). To further verify the regulatory mechanism of necroptosis, we performed unsupervised clustering based on 361 NRSGs to divide the samples into two gene subtypes: gene cluster A and gene cluster B (**Figures 3C, D**). According to the Kaplan-Meier curve, gene cluster B showed a better prognosis (P < 0.001, **Figure 3E**). The heatmap showed the expression distribution of 361 NRSGs in the two gene clusters (**Figure 3F**). Furthermore, significant differences in the expression of NRGs were observed between distinct gene clusters, which was consistent with the expected results of the necroptosis regulatory pattern (**Figure 3G**).

### Characteristics of TME between different gene subtypes

**Figure 4:**
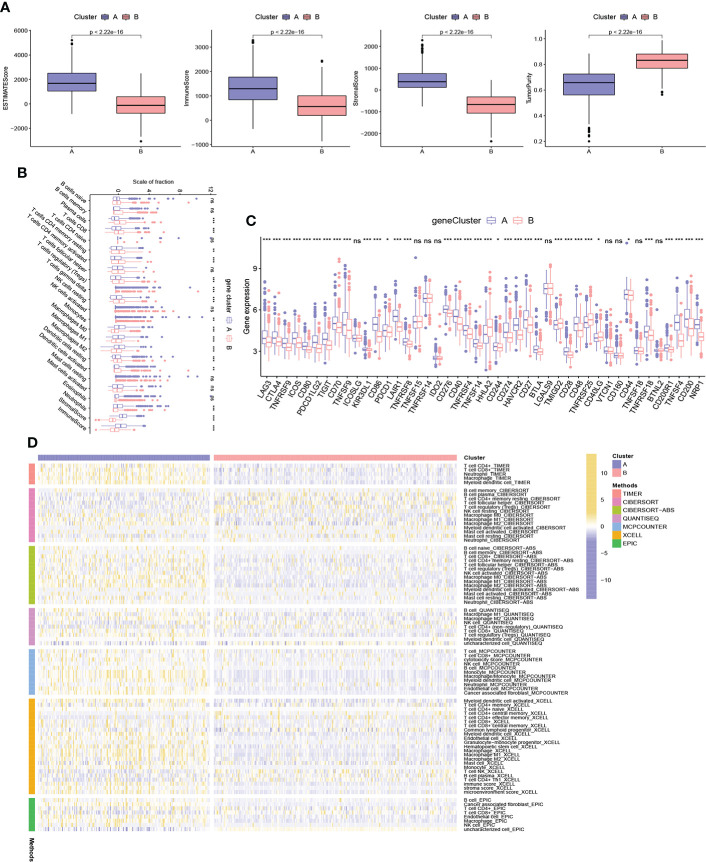
Characteristics of TME between different gene subtypes. **(A)** Estimate scores, stromal scores, immune scores, and tumor purity of two gene subtypes. **(B)** TME immune-infiltrating characteristics and transcriptome traits of two gene clusters. **(C)** Different expression of immune checkpoint genes in distinct gene clusters. **(D)** Heatmap showing differences in immune-infiltrating characteristics between two gene clusters of TCGA samples based on multiple algorithms. ***P < 0.001; **P < 0.01; *P < 0.05; ns P ≥ 0.05.

We assessed the potential role of NRPGs in the tumor microenvironment of CRC. Based on the ESTIMATE algorithm, gene cluster A exhibited higher estimate scores, stromal scores, immune scores, and lower tumor purity (**Figure 4A**). This suggested that the TME of gene cluster A had a higher content of stromal cells and immune cells. Through CIBERSORT algorithm, we further observed that plasma cells, CD8^+^ T cells, naive CD4^+^ T cells, resting and activated CD4 memory cells, Tregs, resting NK cells, and dendritic cells were more abundant in gene cluster B, while M0 macrophages, M1 macrophages, M2 macrophages, and neutrophils were significantly higher in gene cluster A (**Figure 4B**). In addition, we integrated immune cell infiltration information of TCGA samples from multiple existing databases. Through comparison, overall immune cell infiltration was more abundant in gene cluster A (**Figure 4D**). Finally, we compared the expression levels of immune checkpoint genes between the two clusters. The results showed that the vast majority of immune checkpoint genes were highly expressed in subtype A, including the most well-known genes like LAG3, CTLA4, ICOS, TIGIT, PDCD1, CD274 (PD-L1), BTLA (**Figure 4C**). Previous studies have shown that macrophage infiltration is associated with poor prognosis in CRC, whereas infiltration of NK cells, NKT cells, and γδT cells predicts good prognosis (28). Although cluster A generally had higher infiltration of immune cells and stromal cells, it had higher infiltration of macrophages and lower infiltration of CD8^+^ T cells. Besides, the high expression of immune checkpoint genes can cause the depletion of immune cells. These factors may ultimately lead to a worse prognosis of subtype A.

### Construction of NR-signature

A prognostic signature was constructed based on NRSGs. We randomly divided the samples into a training group (n = 569) and a testing group (n = 569). LASSO and Cox analyses were subsequently performed on the 361 NRPGs to further construct the optimal prognostic model (**Supplementary Figure 3A**). Finally, we included 25 genes into the model, and the signature was constructed as follows: NRG riskscore=-0.364*PALLD+0.215*VSIG4+0.329*ANTXR2-0.552*PTPRM+0.269*IHH+0.595*SIGLEC1-0.186*CXCL13-0.509*CEBPA+0.185*PLK2+0.190*EGR2-0.339*IGFBP5-0.567*UBE2L6+0.275*AIFM3+0.303*ARMCX2+0.259*IGFBP3+0.192*ACE2+0.306*HOXC6-0.077*SPINK1+0.146*FABP4-0.296*THBS4-0.175*PHGR1-0.173*MMP12-0.161*CKMT2-0.167*PLCB4+0.278*VIP.

**Figure 5:**
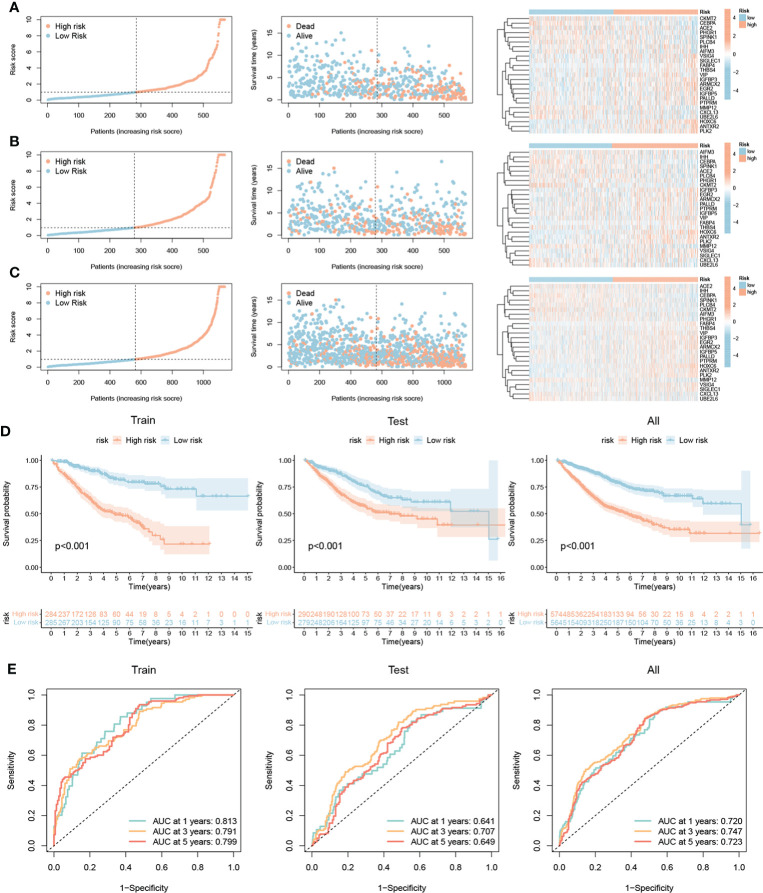
Construction of necroptosis-related signature. Ranked dot and scatter plots showing the risk score distribution and patient survival status, and the heatmap showing differential expression of 25 mRNAs in the high-risk and low-risk groups in the training group **(A)**, testing group **(B)**, and all sample **(C)**. **(D)** Kaplan–Meier analysis of the overall survival between the high and low-risk groups. **(E)** ROC curves to test the prediction performance of signature of 1-, 3-, and 5-year survival according to the risk score.

According to the median value of the risk score of the training group, we divided the patients into a high-risk group and a low-risk group. With the increase in risk scores, the proportion of death among patients also increased. The heatmap showed differential expression of 25 mRNAs in the high-risk and low-risk groups (**Figure 5A**). K-M survival analysis showed that the prognosis of the low-risk group was significantly better than that of the high-risk group (**Figure 5D**). To test the prediction performance of signature, we plotted ROC curves with AUC values of 0.813, 0.791, and 0.799 in the training group at 1, 3, and 5 years (**Figure 5E**). To further validate the signature, we applied it to the testing group and all samples, and the results showed a trend consistent with the training group (**Figures 5B–E**). And we also re-validated the prediction effect of the model in three independent datasets, respectively (**Supplementary Figure 3B, C**).

### Verification and comparison of NR- signature

**Figure 6:**
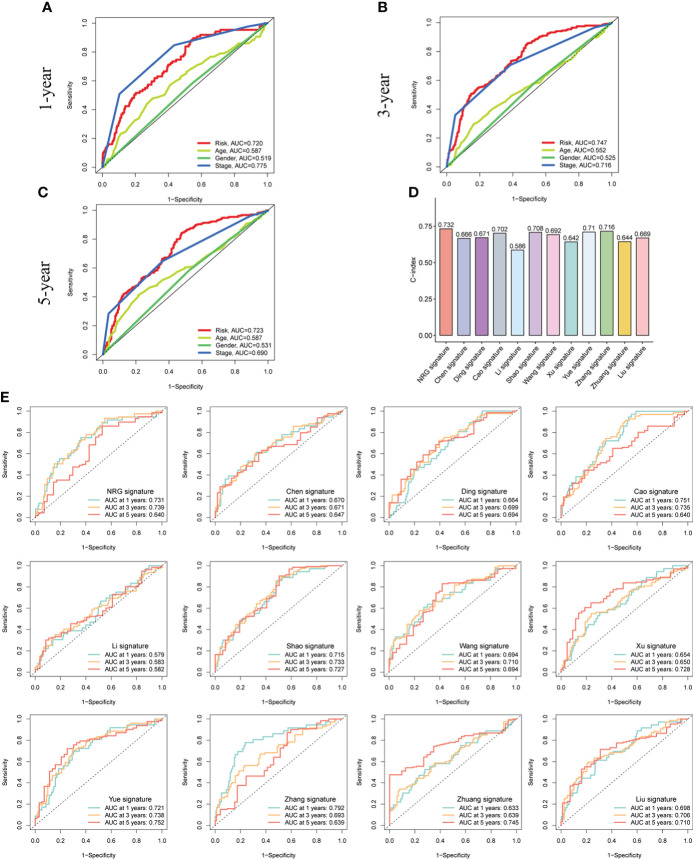
Verification and comparison of the signature. **(A-C)** The comparison of 1-, 3-, and 5-year ROC curves of the signature and other clinical characteristics in the whole cohort. **(D)** C-index of various types of prediction models constructed for CRC. **(E)** ROC curves to compare the prediction performance of various signatures of 1-, 3-, and 5-year survival in the TCGA-COAD cohort.

We compared the predictive performance of the risk model and other clinical indicators. In the prediction of 1-year OS, the effect of the stage was slightly better than that of the risk model, while in the prediction of 3- and 5-year OS, the prediction ability of the risk model was better than other indicators (**Figures 6A–C**). Similarly, we also re-compared each indicator in the three datasets, and the results showed good predictive performance of the risk model (**Supplementary Figure 4**).

In addition, various types of prediction models have been constructed in CRC. To further test the effect of NR-signature, we compared the prediction accuracy of various models based on the TCGA database. By calculating, we found that the C-index of the NR-signature was higher than other types of models (**Figure 6D**). Besides, we also plotted the ROC curve and K-M survival curve of each model (**Figure 6E**; **Supplementary Figure 5**). The results demonstrated that the NR-signature had good prediction performance and can be used as a complement to existing types of prediction models.

### GSEA analysis, mutation, and clinical-related analysis of NR-signature

**Figure 7:**
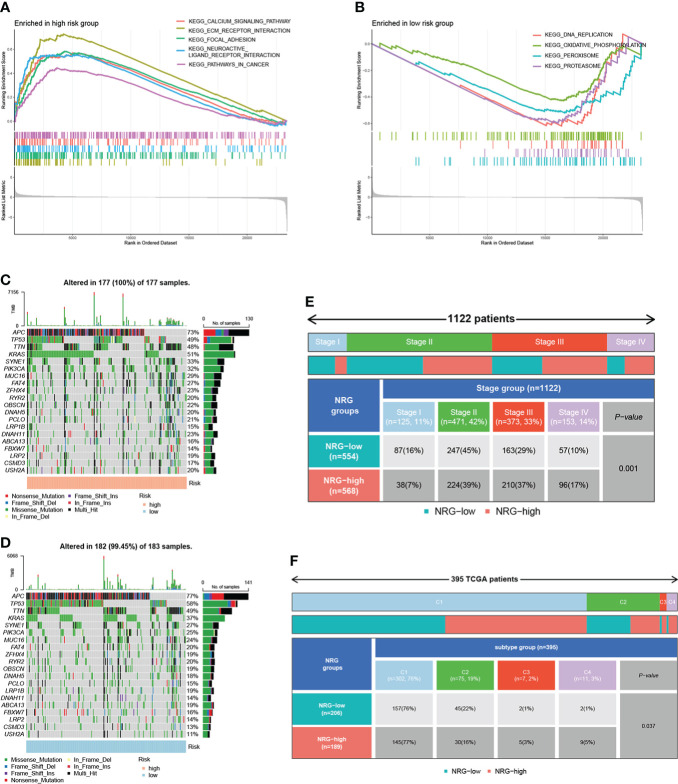
GSEA analysis, mutation, and clinical-related analysis of the signature. GSEA analysis showing pathways enriched in the high-risk group **(A)** and low-risk group **(B)**. The distribution of somatic mutations in the high-risk group **(C)** and low-risk group **(D)**. **(E)** The correlation between risk scores and stage. **(F)** The correlation between risk scores and immune subtypes.

To investigate the underlying biological processes in the high- and low-risk groups, we performed GSEA analysis based on the KEGG geneset. The results showed that tumor-related pathways were enriched in the high-risk group, including ECM RECEPTOR INTERACTION, FOCAL ADHESION, and PATHWAYS IN CANCER. In addition, CALCIUM SIGNALING PATHWAY, which regulates cell survival and death, was also enriched in the high-risk group (**Figure 7A**). In the low-risk group, DNA REPLICATION, OXIDATIVE PHOSPHORYLATION, PEROXISOME, and PROTEASOME were significantly enriched (**Figure 7B**). Next, we analyzed the distribution of somatic mutations in the high and low-risk groups based on TCGA-COAD data, and the top ten mutated genes in both groups were APC, TP53, TTN, KRAS, SYNE1, PIK3CA, MUC16, FAT4, ZFHX4, and RYR2 (**Figures 7C, D**). APC and TP53 are the most widespread and representative mutations in colorectal cancer, and detecting the mutation status of these genes can help improve the accuracy of diagnosis and guide individualized treatment. Moreover, several studies have focused on reactivating TP53 function to exert its anti-tumor effects (29, 30). Among the mutated genes, the mutation rate of KRAS was significantly higher in the high-risk group than that in the low-risk group (51% vs. 37%, P = 0.009). According to studies, the prevalence of KRAS mutation in colorectal cancer is about 40%, and KRAS mutation suggests poor prognosis (31–33). The 8th AJCC Cancer Staging Manual specifies that the level of evidence for KRAS mutation as a prognostic and predictive factor is grade I and II, respectively (33). In addition, KRAS gene mutation indicates poor response of colorectal cancer patients to anti-EGFR targeted therapy (32, 33). The higher frequency of KRAS mutations in the high-risk group in our analysis is consistent with these findings. Additionally, we assessed the correlation of risk scores with clinical indicators. The proportion of patients with high and low risk varied by stage, with fewer high-risk patients than low-risk patients within stage I-II and more high-risk patients within stage III-IV (**Figure 7E**). Moreover, the proportion of patients with high and low risk differed across immune subtypes (**Figure 7F**).

### Assessment of TME and immune checkpoints in high- and low-risk groups

**Figure 8:**
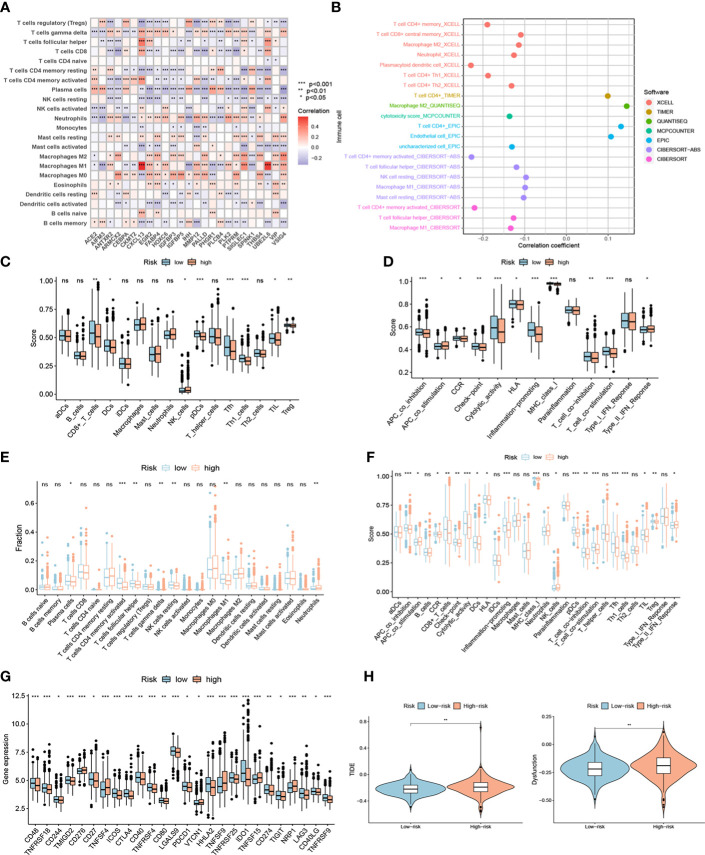
The immune landscape of high- and low-risk groups. **(A)** Heatmap showing a close correlation between genes in the signature and immune cells. **(B)** Correlation analysis showed that the overall risk score was negatively correlated with immune infiltration in TCGA samples. Different contributions of immune cell infiltration and immune function in distinct risk groups based on ssGSEA algorithm **(C-D)** and CIBERSORT algorithm **(E-F)**. **(G)** Different expression of immune checkpoint genes in high- and low-risk groups. **(H)** TIDE scores and Dysfunction scores of distinct risk groups based on TCGA samples. ***P < 0.001; **P < 0.01; *P < 0.05; ns P ≥ 0.05.

Based on the CIBERSORT algorithm, the heatmap showed a close correlation between the 25 genes in NR-signature and immune cells (**Figure 8A**). We explored the relationship between risk score and immune cells of TCGA samples according to immune cell infiltration information from multiple databases. Correlation analysis showed that the overall risk score was negatively correlated with immune infiltration (**Figure 8B**). We then assessed immune cell infiltration and immune function in all samples using different methods. ssGSEA analysis showed that the infiltration of CD8^+^ T cell, DC (dendritic cell), pDC (plasmacytoid dendritic cells), Tfh (follicular T-helper-cell), Th1, TIL (tumor-infiltrating lymphocytes) and Treg was lower in the high-risk group, and APC (antigen-presenting cell) co-inhibition, cytolytic activity, inflammation-promoting, T cell functions such as co-inhibition and co-stimulation were attenuated (**Figures 8C, D**). The CIBERSORT algorithm showed a similar trend, with lower immune infiltration and immune function overall in the high-risk group, and a significant decrease in CD8^+^ T cells, cytolytic activity, inflammation-promoting, and TIL (**Figures 8E, F**). We also performed a survival analysis of these immune cells and functions, and low-grade infiltration of the aforementioned important immune cells and functions was associated with poor prognosis (**Supplementary Figure 6**). The expression levels of multiple immune checkpoint genes also differed between high and low-risk groups (**Figure 8G**). Finally, we calculated TIDE scores and Dysfunction scores based on samples from the TCGA database, and the results showed that patients in the high-risk group scored higher, predicting a higher potential for immune escape and immune dysregulation (**Figure 8H**).

### Pan-cancer analysis of 25 genes included in NR-signature

To further validate the functions of the genes included in NR-signature, we explored these genes in pan-cancer based on the TCGA database. First, we compared the expression levels of 25 genes in cancer tissues and normal tissues, and the results showed that 25 genes showed significant expression differences in multiple cancer types (**Supplementary Figures 7, 8**). In addition, we also investigated the association of genes with immune microenvironment score and Stemness Score. Among them, VSIG4, SIGLEC1, and CXCL13 were significantly positively correlated with estimate scores, stromal scores, and immune scores in pan-cancer, while negatively correlated with tumor purity (**Supplementary Figure 9**). In general, genes were negatively correlated with RNAss. Subsequently, we analyzed the relationship between genes and immune subtypes in COAD, and boxplots indicated differences in gene expression among different immune subtypes (**Supplementary Figure 10**). We also re-analyzed the association of 25 genes with immune microenvironment score and stemness score in COAD, and the expression of most genes showed a strong positive correlation with the immune microenvironment score (**Supplementary Figure 11**).

### Kaplan-Meier survival analysis and expression information of genes in NR-signature

**Figure 9:**
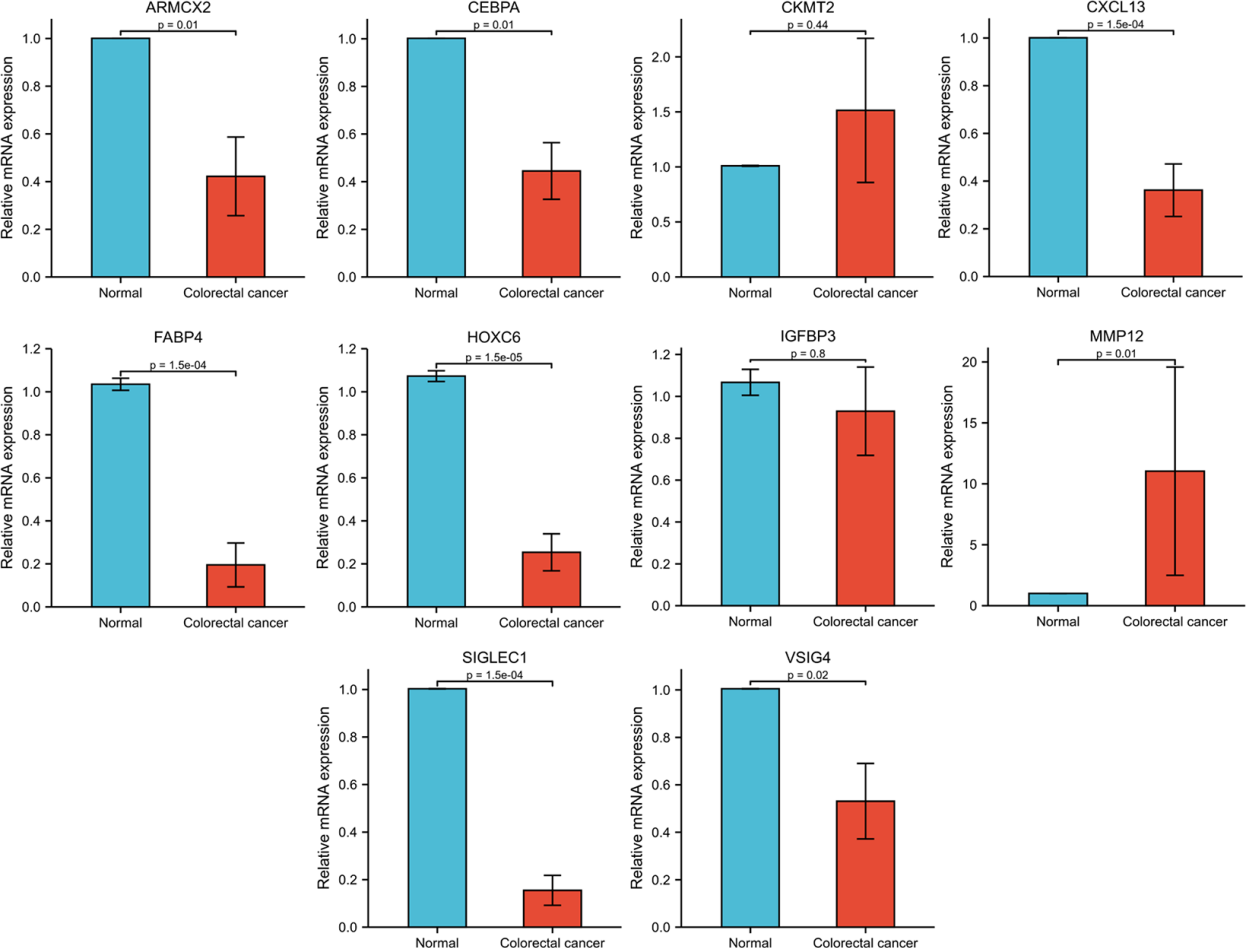
Expression information of genes in NR-signature based on CRC and normal tissues.

To further validate the functions of the genes included in the model, we first performed KM survival analysis of these genes based on the TCGA database (**Supplementary Figure 12**), and selected 10 survival-related genes for expression validation. RT-qPCR results based on eight pairs of colorectal cancer tissues showed that eight of the 10 genes were significantly differentially expressed between cancer and normal tissues. Among them, MMP12 was highly expressed in colorectal cancer tissues, while ARMCX2, CEBPA, CXCL13, FABP4, HOXC6, SIGLEC1 and VSIG4 were more expressed in normal tissues than in cancer tissues (**Figure 9**).

## Discussion

Recently, a large number of studies have revealed the important regulatory role of necroptosis in tumor progression and immune response. Han et al. revealed that Resibufogenin inhibits CRC growth and metastasis through RIP3-mediated necroptosis (34). Hsieh et al. showed that carnosine can inhibit human colorectal cancer cell proliferation by inducing necroptosis, autophagy, and reducing angiogenesis (35). However, most of the current studies have focused on the role of necroptosis mediated by specific molecules in tumors, the overall effect exerted by the combined action of multiple NRGs and the comprehensive landscape of immune infiltration in TME have not been fully elucidated. There have also been many studies investigating the overall role of certain classes of genes and their regulation of the tumor immune microenvironment based on different sets of functional genes in different cancer species, such as costimulatory molecule-related genes, RNA-n6-methyladenosine-related genes, ferroptosis-related genes, etc (36–39). Therefore, clarifying the overall changes in NRGs at the transcriptional and genetic levels and the characteristics of immune cell infiltration in the TME will help us to deepen our understanding of anti-tumor immune responses and provide new insights for promoting more accurate risk stratification and clinical treatment for patients.

Through differential expression analysis, we found that MLKL was highly expressed in CRC tissues, while RIPK1 and RIPK3 were lowly expressed in cancer tissues. The result is consistent with previous findings. In a panel of more than 60 cancer cell lines, two-thirds of the cells had reduced levels of the RIPK3 protein, and cancer cells tended to escape necrosis to survive (40). Survival analysis also showed that low expression of RIPK3 was closely associated with poor prognosis. We identified two NRG subtypes based on 67 NRGs, with cluster A showing a better prognosis. GSVA analysis revealed a significant enrichment of immune-related pathways such as B cell receptor and T cell receptor signaling pathways in cluster B. There were also significant differences in TME characteristics between the two clusters, with cluster B showing more abundant immune cell infiltration. In NRG cluster B, the contents of activated CD8^+^ T cells, activated CD4^+^ T cells, Tfh, and activated B cells were higher, but the regulatory T cells and MDSCs which play an immunosuppressive role were also infiltrated in large numbers. These immune effector cells are regulated by immune regulatory cells, regulatory molecules, and immune checkpoints. Treg cells infiltrating locally in tumors can inhibit the proliferation and activation of T cells, and combine with MDSCs and tumor-associated macrophages to form an immunosuppressive network and participate in the formation of tumor immune tolerance (41–43). This indicated that under the long-term stimulation of tumor antigens, T cells, especially CD8^+^ T cells, may be inhibited by Tregs, with their effector function in a depleted state which can not effectively kill tumor cells. In addition, immune checkpoint gene expression was generally significantly upregulated in subtype B, including the inhibitory molecules PDCD1, CTLA4, LAG3, and HAVCR2 (TIM3) which are highly expressed by depleted T cells.

In addition, mRNA transcriptome differential expression genes between distinct NRG subtypes were closely related to biological processes such as immune-related processes, cytokines, chemokines, and tumor signaling pathways. We identified two gene subtypes based on DEGs associated with prognosis between NRG subtypes. Survival analysis indicated that gene subtypes B had a better prognosis. Although subtype B had a lower immune microenvironment score, with fewer immune cells and stromal cells overall than subtype A, it contained abundant infiltrating effector T cells such as CD8^+^ T cells and CD4^+^ memory cells, accompanied by lower immune checkpoint gene expression. It has been shown that high levels of PD-1 (PDCD1) expression correlate with increased numbers of tumor-infiltrating Tregs and decreased numbers of effector T cells in the TME of CRC, which can be reversed by immune checkpoint blockades (ICB) (44, 45). Our analysis showed a partially similar trend, with gene cluster A having higher PDCD1 expression accompanied by lower levels of infiltrating CD8^+^ T cells and Tregs cells. Several studies have reported that PD-1/PD-L1 (CD274) expression levels in cancer patients provide important predictive information for assessing sensitivity to ICBs before treatment (46–48). A study by Chen et al. showed that CRC patients with high PD-L1 expression responded more effectively to ICBs (49). This suggests that for gene cluster A with higher PD-1 and PD-L1 expression and lower infiltration of CD8^+^ T cells, ICB may have higher sensitivity to reverse the state of the tumor microenvironment and improve prognosis. In addition, according to the literature, PD-L1 expression in cancer cells can promote macrophage recruitment, and macrophages induce PD-L1 upregulation and suppress CD8^+^ T cell responses, which in turn promote macrophage infiltration, forming a positive feedback loop that promotes immune escape of cancer cells and thus CRC growth (50). The above findings also support the results of our analysis that gene cluster A with high PD-L1 expression and macrophage infiltration had a poor prognosis.

The above findings suggested that NRGs can be used as an effective indicator for assessing clinical survival and immune infiltration in CRC. Therefore, we further construct the NR-signature, and the model exhibited robust and effective prediction ability. Compared with other clinical indicators and published prediction models, the good performance of our model showed that NR-signature can act as a supplement to the existing research. Patients in the high-risk and low-risk groups showed different clinical characteristics, survival time, immune infiltration, and expression of immune checkpoints. The high-risk group had lower CD8^+^ T cells, TIL, and Tfh cells, with a low state of many immune functions such as T cell co-activation and co-suppression, which may lead to immune dysfunction and increased risk of immune escape. The low-risk group was relatively immune-activated, although most immune checkpoint genes were highly expressed. This suggested that the application of immune checkpoint inhibitors in the low-risk group may have better results, which can further enhance the original anti-tumor immune effect. Overall, NR-signature can be used for prognostic stratification of patients, guide more effective immunotherapy, and contribute to a deeper understanding of the underlying molecular mechanisms of CRC.

Among the genes selected to construct the NR-signature, VSIG4, SIGLEC1, and CXCL13 showed significant correlations with immune microenvironment scores in pan-cancer. VSIG4 (V-set and immunoglobulin domain–containing 4) is a complement receptor of the immunoglobulin superfamily and is specifically expressed in resting tissue-resident macrophages (51, 52). By binding to unrecognized receptors on T cells, VSIG4 can inhibit T cell proliferation and promote the differentiation of Foxp3^+^ Tregs (53). Several other studies have revealed that VSIG4 can negatively regulate the inflammatory response mediated by macrophages (54, 55). Yuan et al. used soluble VSIG4 as a surrogate marker of activated macrophages for the diagnosis of patients with Lymphoma-associated haemophagocytic lymphohistiocytosis (56). Hall et al. believed that VSIG4 is a new marker of adipose tissue macrophages in aged mice and can be used as a biomarker of aging adipose tissue in mice (57). SIGLEC1, sialic acid binding Ig-like lectin 1, is involved in initial contact with sialylated pathogens and mediates phagocytosis and endocytosis of pathogens, thereby promoting immune responses to limit infection (58). It has been reported that SIGLEC1 is abnormally expressed in various autoimmune diseases and can serve as a potential clinical marker, such as monogenic interferonopathies (59), systemic lupus erythematosu (60–62), autoimmune congenital heart block (63), and primary Sjögren’s syndrome (64). CXCL13 is a chemokine targeting B lymphocytes, and its receptor CXCR5 is expressed in specific T lymphocyte subsets (65). Accumulating evidence indicates the important roles of CXCL13 and CXCR5 in the regulation of tumorigenesis, progression, metastasis, and prognosis in the tumor microenvironment (66–68). CXCL13 has prognostic value for CRC patients, and high expression of CXCL13 can also lead to resistance to 5-Fluorouracil (69). These studies have shown that necroptosis-related signature genes such as VSIG4, SIGLEC1, and CXCL13 are closely related to immune activity in the microenvironment, which also reflects the potential interaction between necroptosis and tumor immune regulation. In addition, we also verified the expression of some genes (ARMCX2, CEBPA, CKMT2, CXCL13, FABP4, HOXC6, IGFBP3, MMP12, SIGLEC1, and VSIG4) in the model based on 8 pairs of colorectal cancer tissues. 8 out of 10 genes (ARMCX2, CEBPA, CXCL13, FABP4, HOXC6, MMP12, SIGLEC1, and VSIG4) showed significant differences in expression between cancer and normal tissues. The functional mechanism of ARMCX2 has not been elucidated in detail. The role of CEBPA in hematologic tumors has been extensively studied, but its potential function in CRC remains to be explored (70, 71). CXCL13 (72), FABP4 (73, 74), and HOXC6 (75, 76) have been reported to be highly expressed in CRC and play a pro-tumor role in some studies. However, the gene expression trends we analyzed were reversed and more in-depth studies are needed to clarify the functions of these molecules. It has been shown that MMP12 is highly expressed in colon cancer patients and predicts a poor prognosis (77), which is consistent with the results of our qPCR. We have discussed SIGLEC1 and VSIG4 in various diseases above, but their role in CRC still needs further investigation. Due to insufficient sample size, further experiments are needed to explore the function of these genes in colorectal cancer.

There are some limitations of our study. First, most of the data used for the analysis were derived from public databases. Although our analysis was based on a large sample of more than 1000 cases, these cases were obtained retrospectively, and selection bias in the dataset may also affect the accuracy of the results. Large-scale prospective studies and *in vivo*, *in vitro* mechanistic studies are still needed to further confirm our results. In addition, some important clinical variables such as surgery, chemoradiotherapy, and immunotherapy information are missing in most of the datasets, we also need to combine more clinical characteristics to improve the prediction accuracy.

In conclusion, our comprehensive analysis of NRGs revealed their extensive involvement in the regulatory mechanisms of the tumor immune microenvironment, clinicopathological features, and prognosis. We also constructed a novel signature to comprehensively evaluate the prognostic risk and immune infiltration characteristics of a single case. These findings demonstrated the important clinical implications of NRGs and provided new insights for individualized targeted therapy and immunotherapy of cancer.

## Data availability statement

The original contributions presented in the study are included in the article/**Supplementary Material**. Further inquiries can be directed to the corresponding authors.

## Ethics statement

The studies involving human participants were reviewed and approved by Ethics Committee of Tongji Medical College. Written informed consent for participation was not required for this study in accordance with the national legislation and the institutional requirements.

## Author contributions

MS, LX, and WH designed the study. MX, XJ, XL, and YF collected the literature. MS analyzed the data. DL and YW assisted in analyzing the data. MS, MX, XJ, XL, and YF drafted the manuscript. XC, BZ, YL, BL, LX, and WH modified the manuscript. All authors read and approved the final manuscript.

## Funding

The design of the study and collection, analysis, and interpretation of data and writing the manuscript were supported by the National Natural Science Foundation of China No. 81871911 (WH), No. 82173313 (WH), No. 82273310 (LX), and No. 81972237 (LX).

## Acknowledgments

We acknowledge the database TCGA and GEO. Besides, we are very grateful to Professor Wang Guihua for providing colorectal cancer tissues.

## Conflict of interest

The authors declare that the research was conducted in the absence of any commercial or financial relationships that could be construed as a potential conflict of interest.

## Publisher’s note

All claims expressed in this article are solely those of the authors and do not necessarily represent those of their affiliated organizations, or those of the publisher, the editors and the reviewers. Any product that may be evaluated in this article, or claim that may be made by its manufacturer, is not guaranteed or endorsed by the publisher.

## Abbreviations

NRGs, necroptosis-related genes; CRC, colorectal cancer; TME, tumor microenvironment; TCGA, the Cancer Genome Atlas; GEO, Gene-Expression Omnibus; FPKM, Fragments per Kilobase Million; TPM, Transcripts per million; CDF, cumulative distribution function; GSVA, gene set variation analysis; DEGs, differentially expressed genes; GO, Gene Ontology; KEGG, Kyoto Encyclopedia of Genes and Genomes; ssGSEA, single sample GSEA; GSEA, Gene Set Enrichment Analysis; lasso, least absolute shrinkage and selection operator; OS, overall survival; ROC, receiver operating characteristic; C-index, concordance index; TMB, tumor mutational burden; MDSC, Myeloid-derived suppressor cell; Treg, Regulatory T cell; NRSGs, necroptosis-related signature genes; DC, dendritic cell; pDC, plasmacytoid dendritic cells; Tfh, follicular T-helper-cell; TIL, tumor-infiltrating lymphocytes; APC, antigen-presenting cell; ICB, immune checkpoint blockades.
