# Supplementary material for: Identification of necroptosis-related subtypes, development of a novel signature, and characterization of immune infiltration in colorectal cancer
Source: Front Immunol. 2022 Dec 5;13:999084. doi: 10.3389/fimmu.2022.999084 (PMC9762424; doi:10.3389/fimmu.2022.999084)
Supplement: Supplementary file 1 [file DataSheet_1.docx]

Supplementary Material


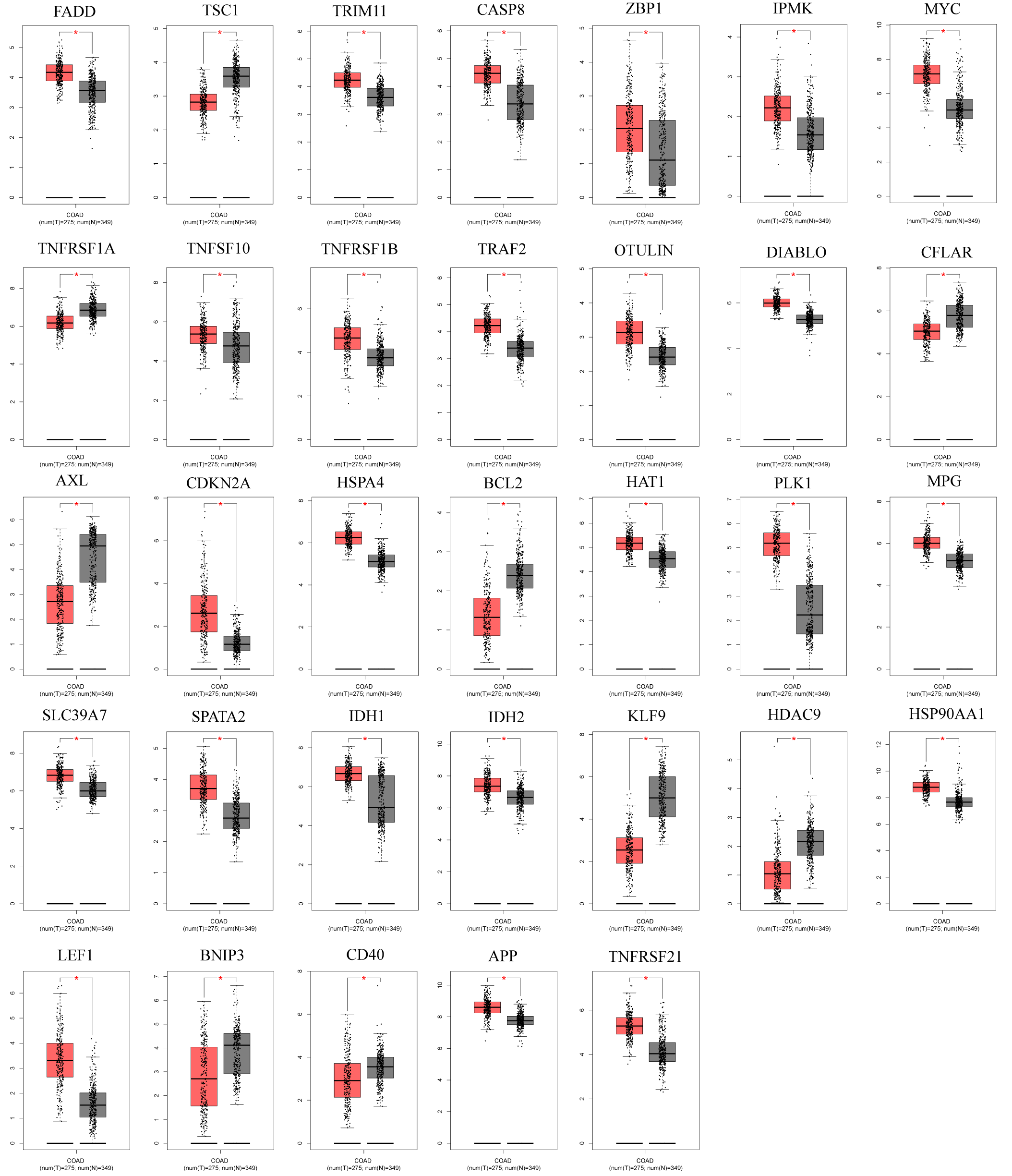


**Supplementary Figure 1. 33 differentially expressed NRGs (TCGA combined with GTEx).**


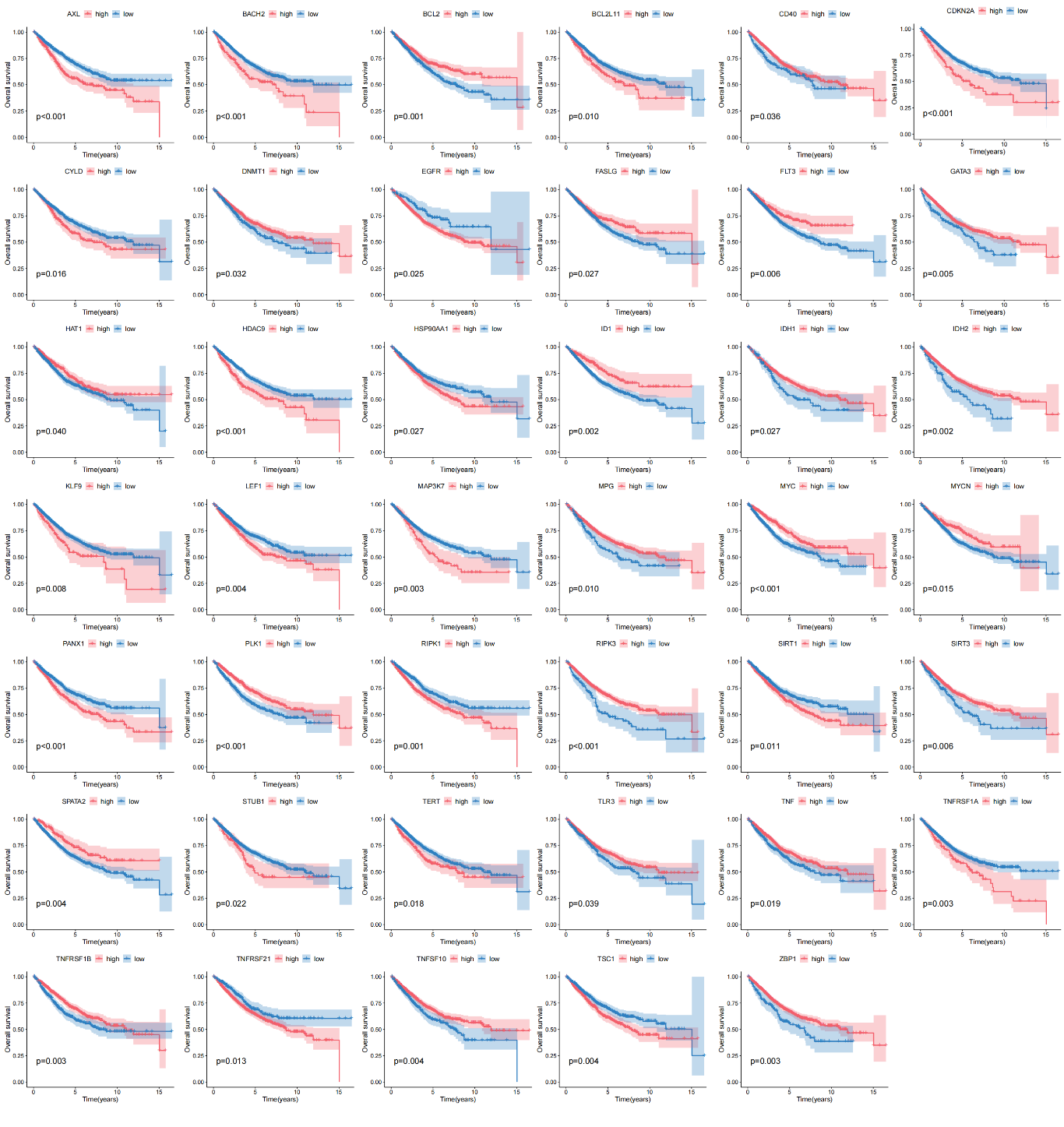


**Supplementary Figure 2.** Kaplan-Meier survival analysis of 67 necroptosis-related genes.


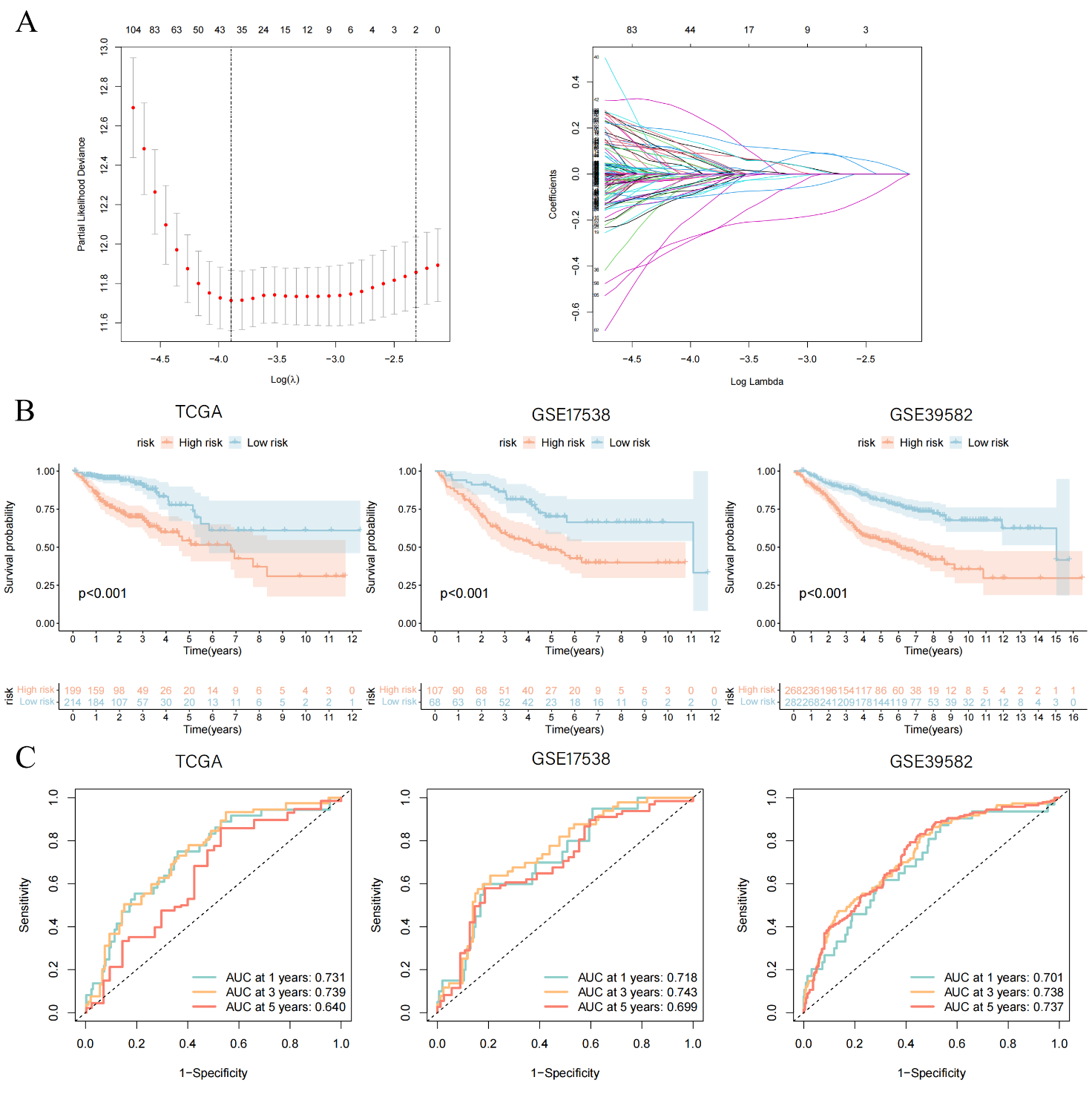


**Supplementary Figure 3.** (A) Selection of the optimal parameter (lambda) in the LASSO model and LASSO coefficient profiles. (B) K–M analysis of the overall survival between the high and low-risk groups in three independent datasets. (C)The prediction effect of the model in three independent datasets.


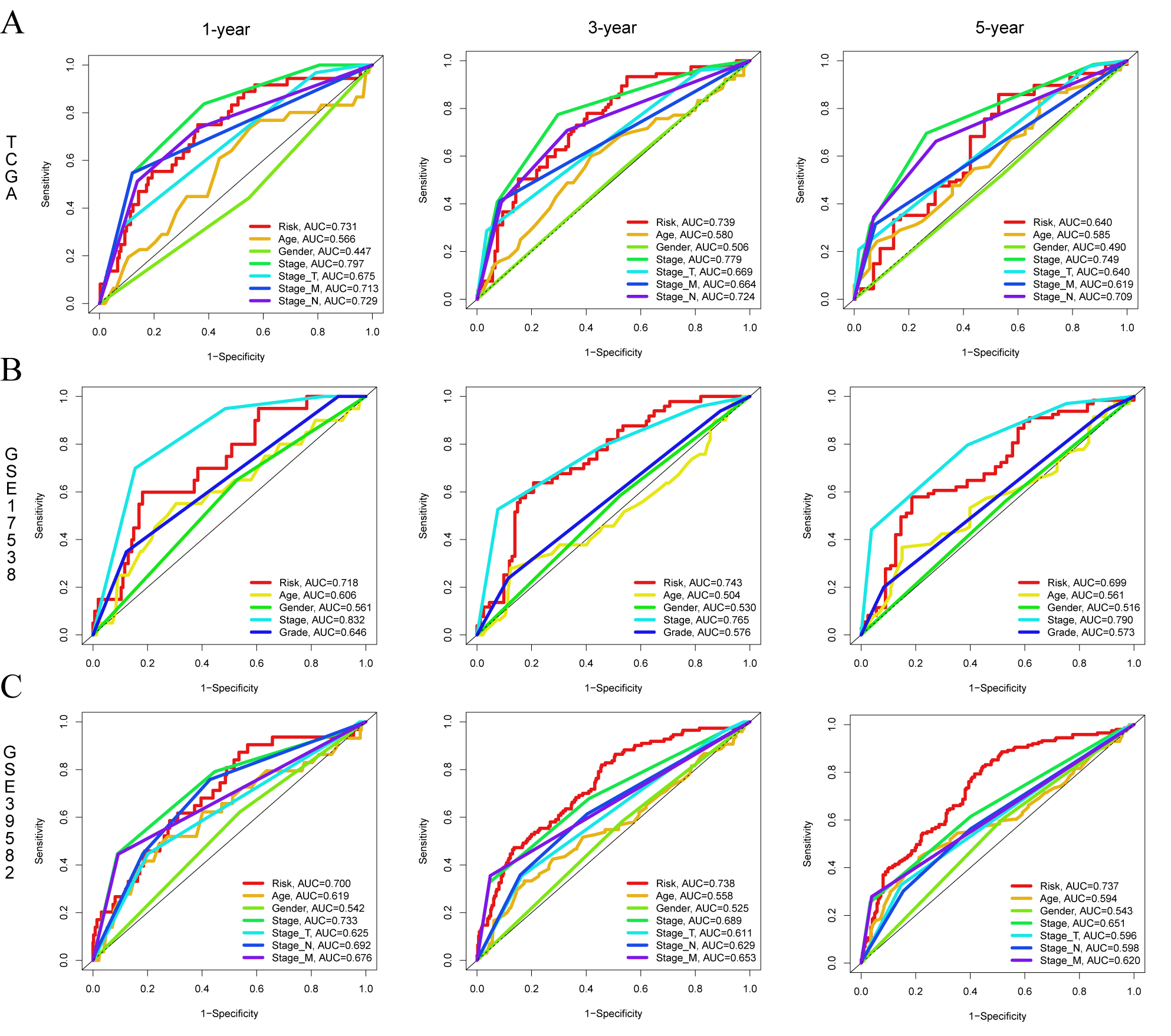


**Supplementary Figure 4.** The comparison of the predictive performance of the risk model and other clinical indicators in three independent datasets.


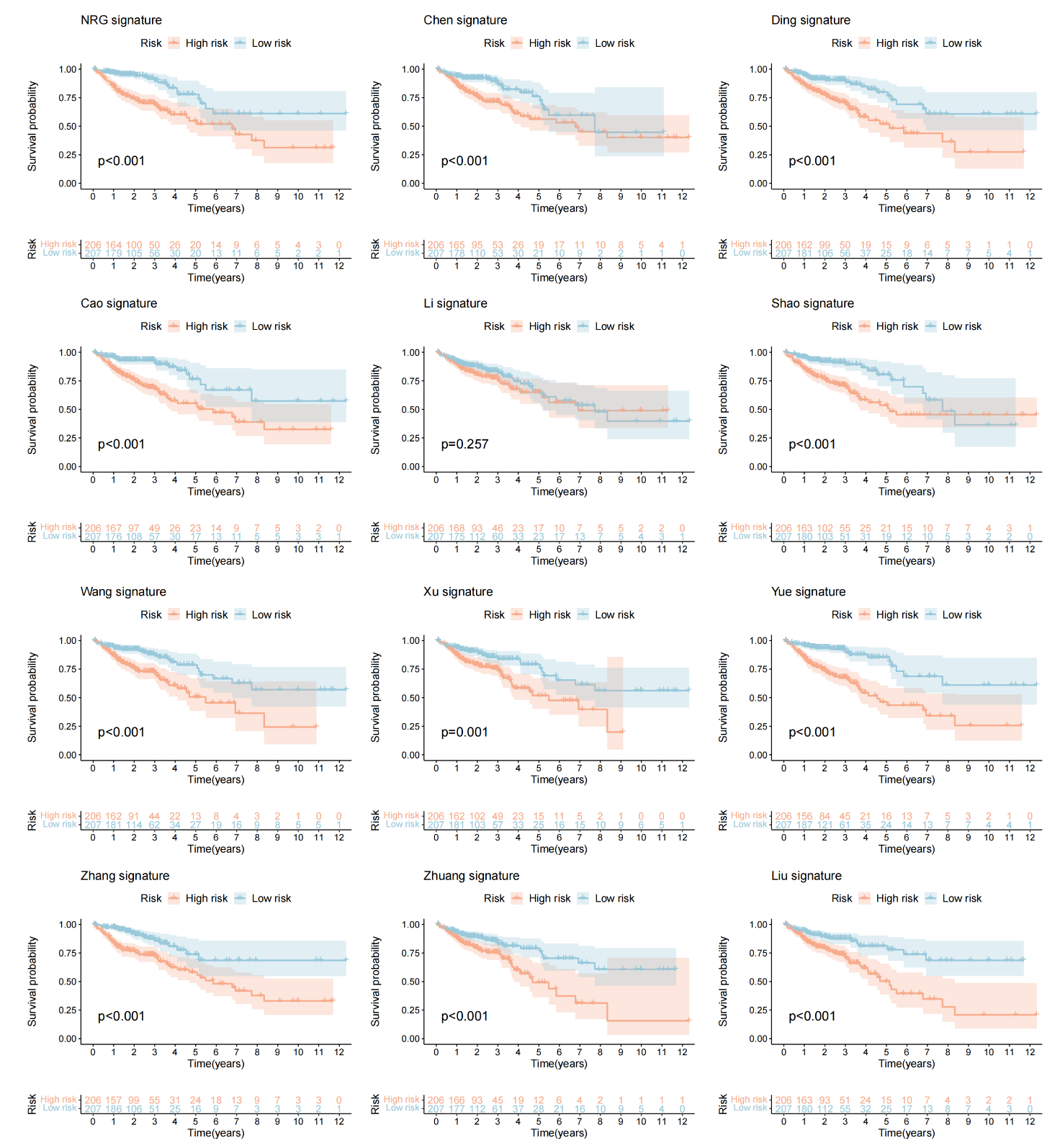


**Supplementary Figure 5.** K–M analysis of various types of prediction models constructed for CRC.


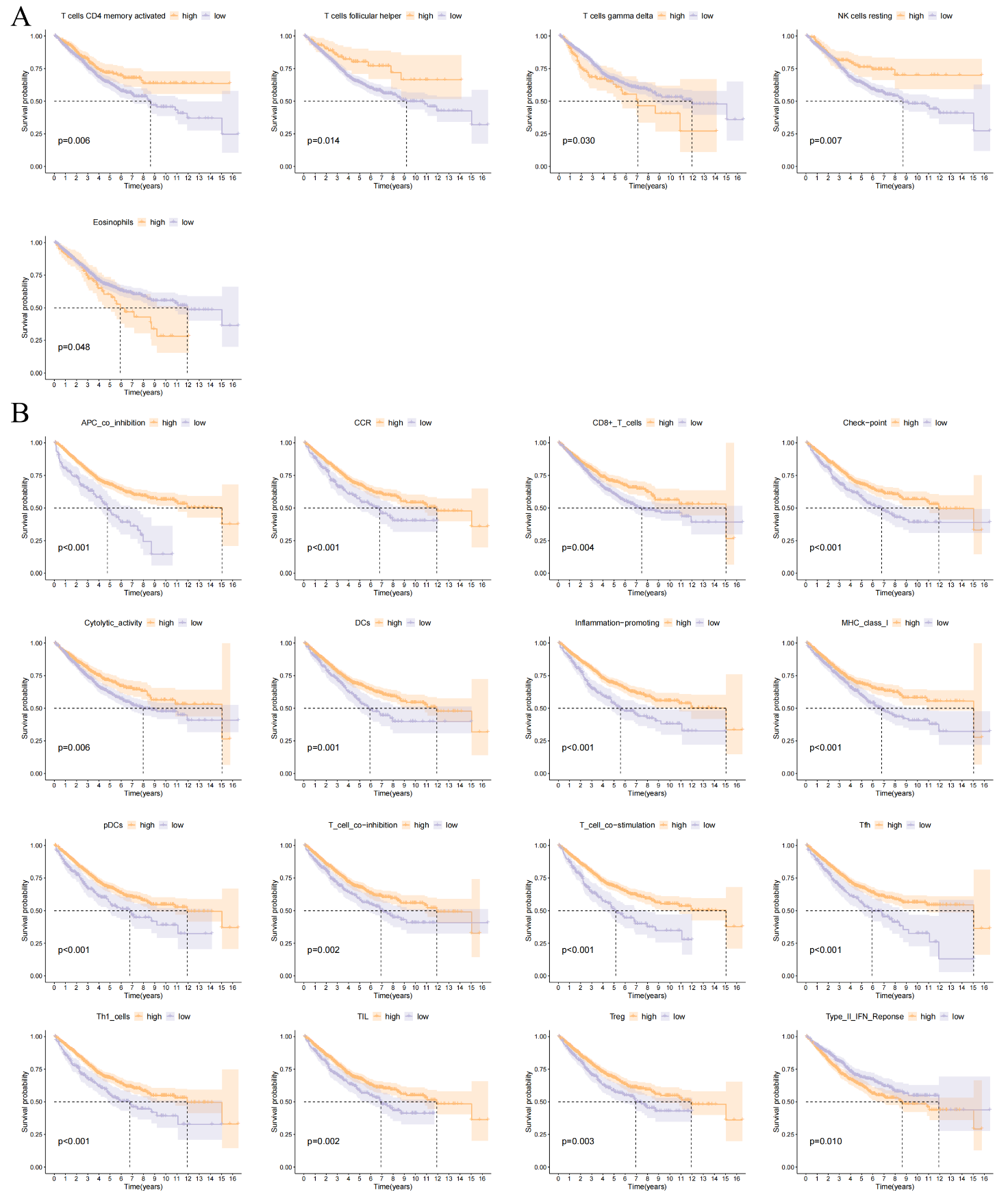


**Supplementary Figure 6.** Survival analysis of infiltrating immune cells and functions.


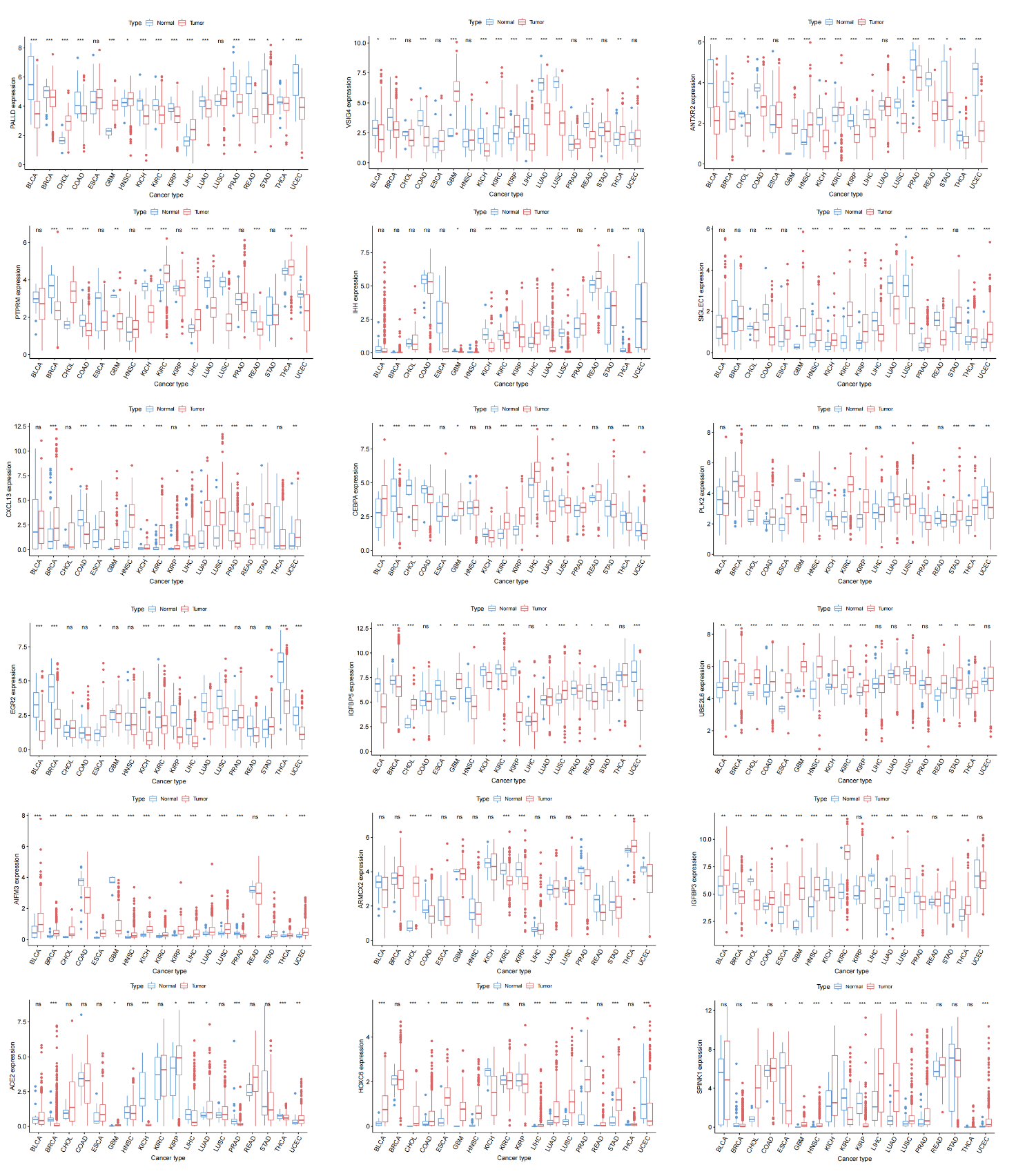


**Supplementary Figure 7.** Expression differences of genes included in the signature in pan-cancer (18 genes).


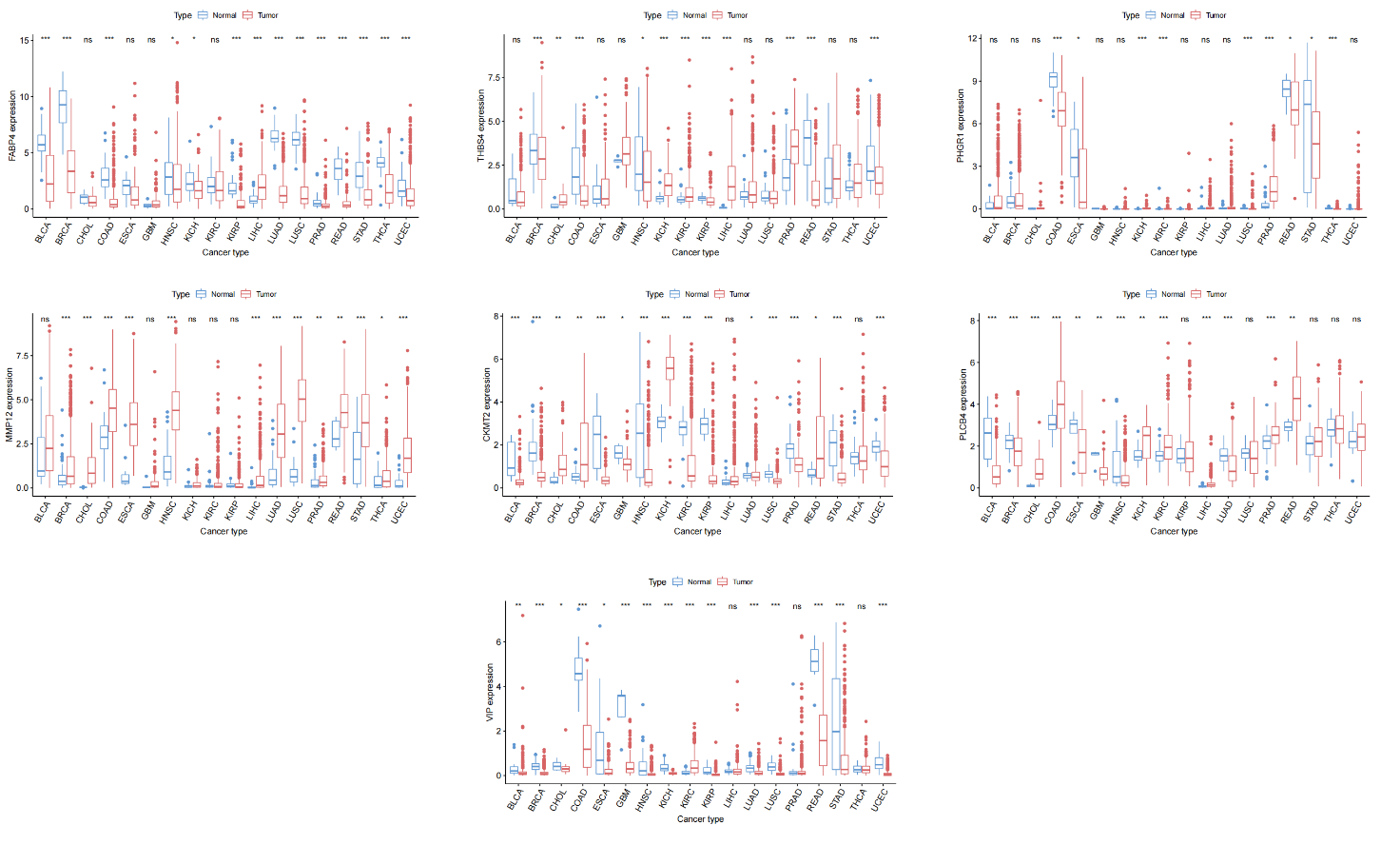


**Supplementary Figure 8.** Expression differences of genes included in the signature in pan-cancer (7 genes).


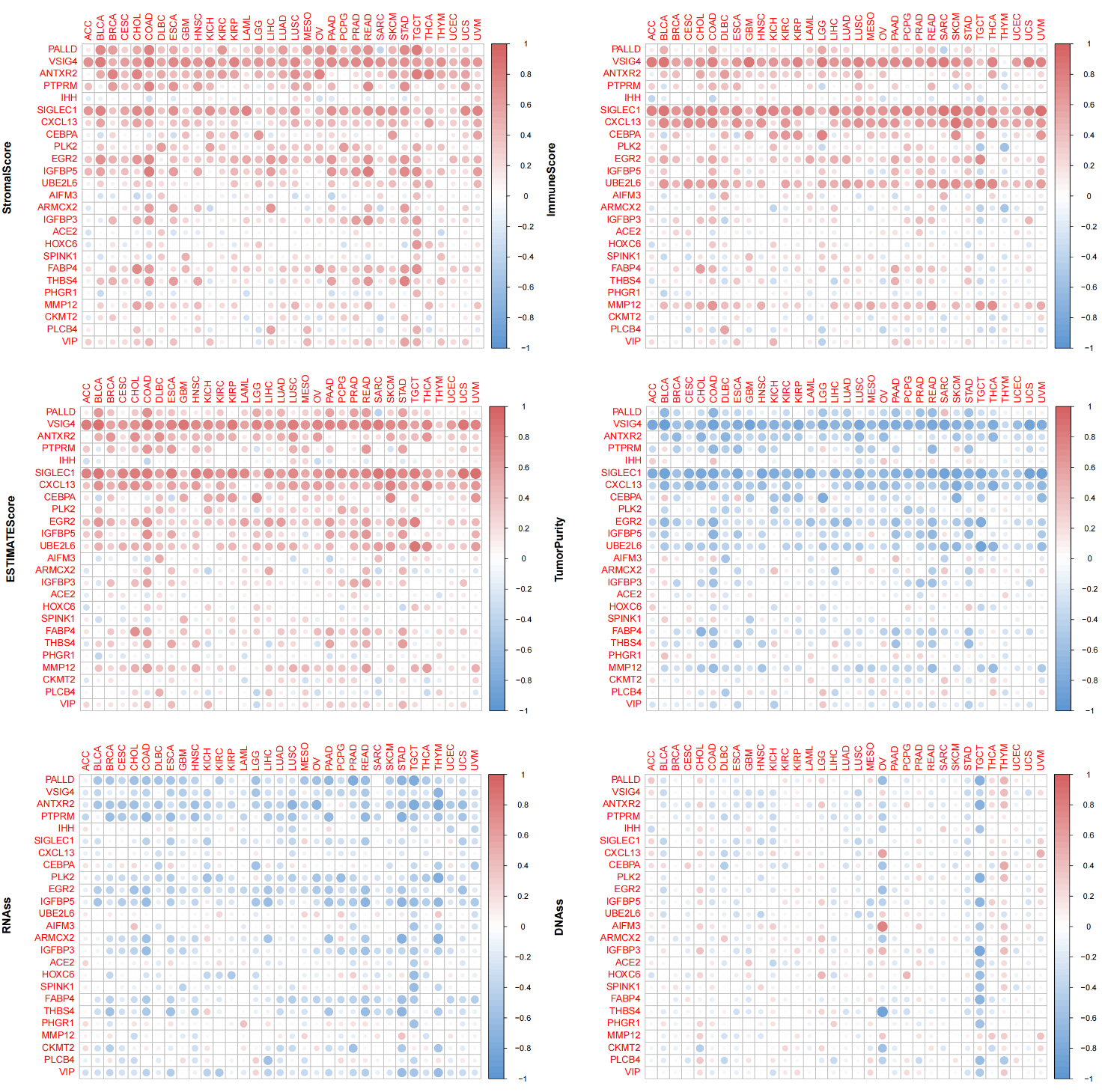


**Supplementary Figure 9.** The association of genes included in the signature with immune microenvironment score and Stemness Score.


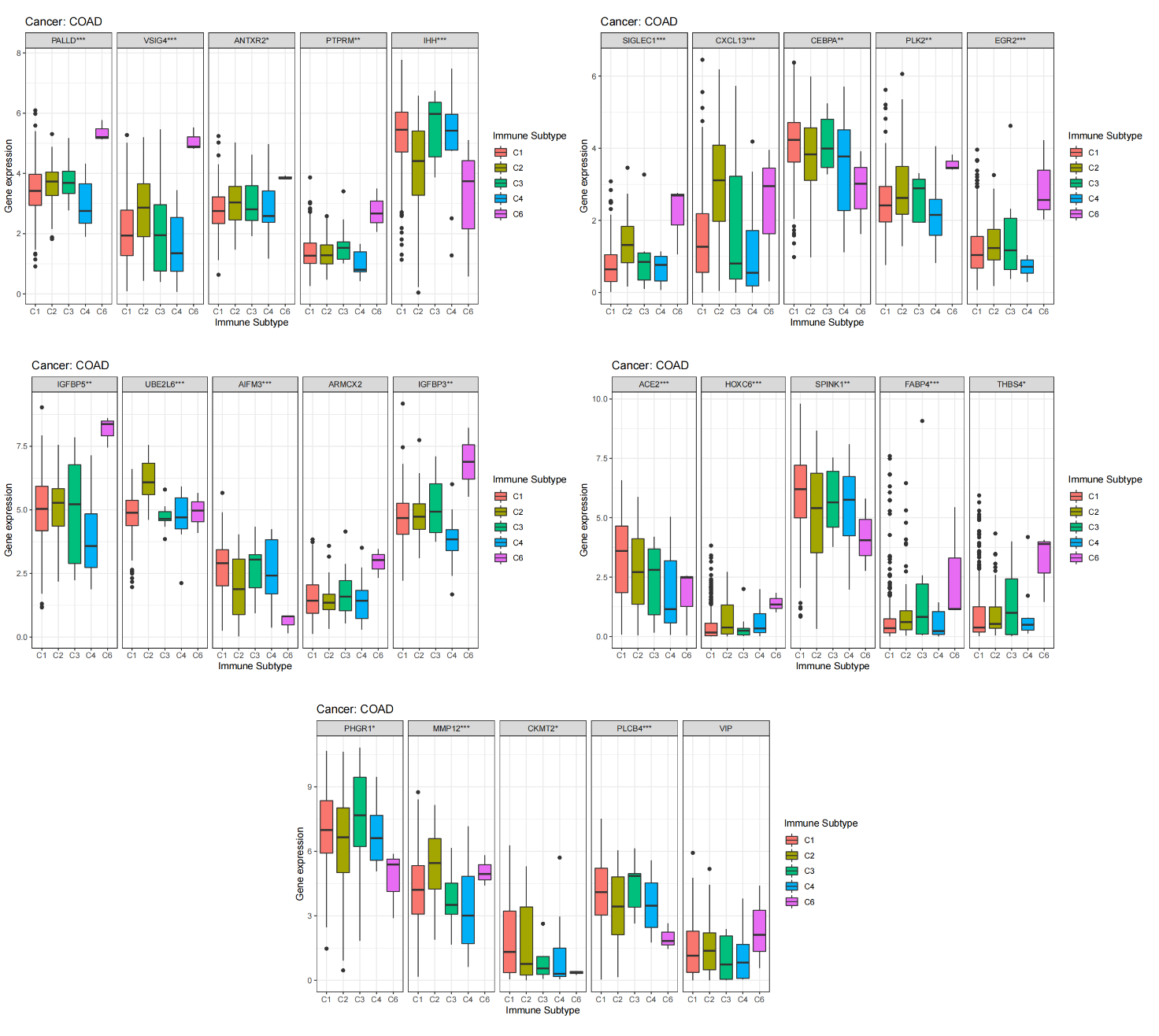


**Supplementary Figure 10.** Differences in the expression of genes included in the signature among different immune subtypes.


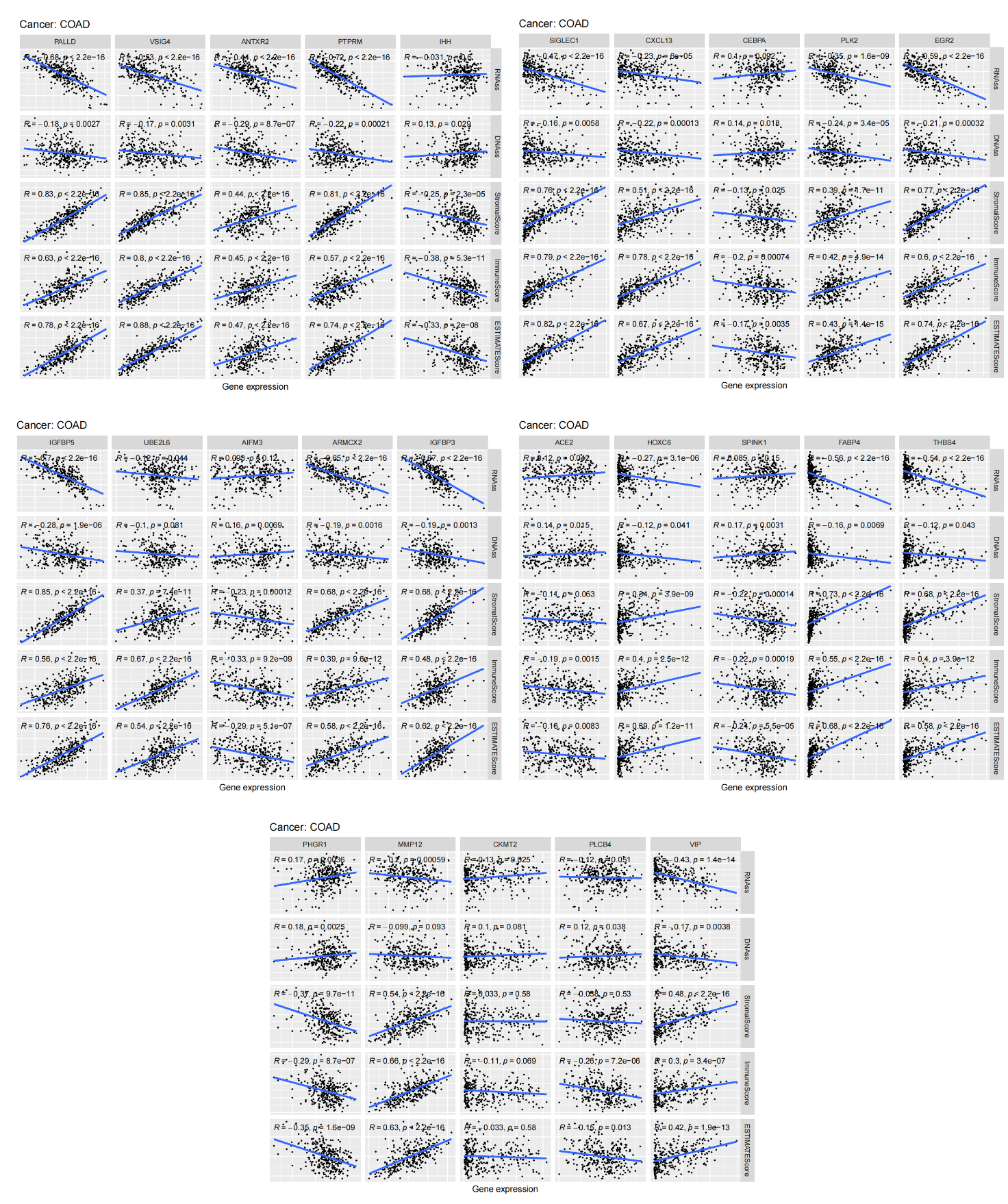


**Supplementary Figure 11.** The association of genes included in the signature with immune microenvironment score and stemness score in CRC.


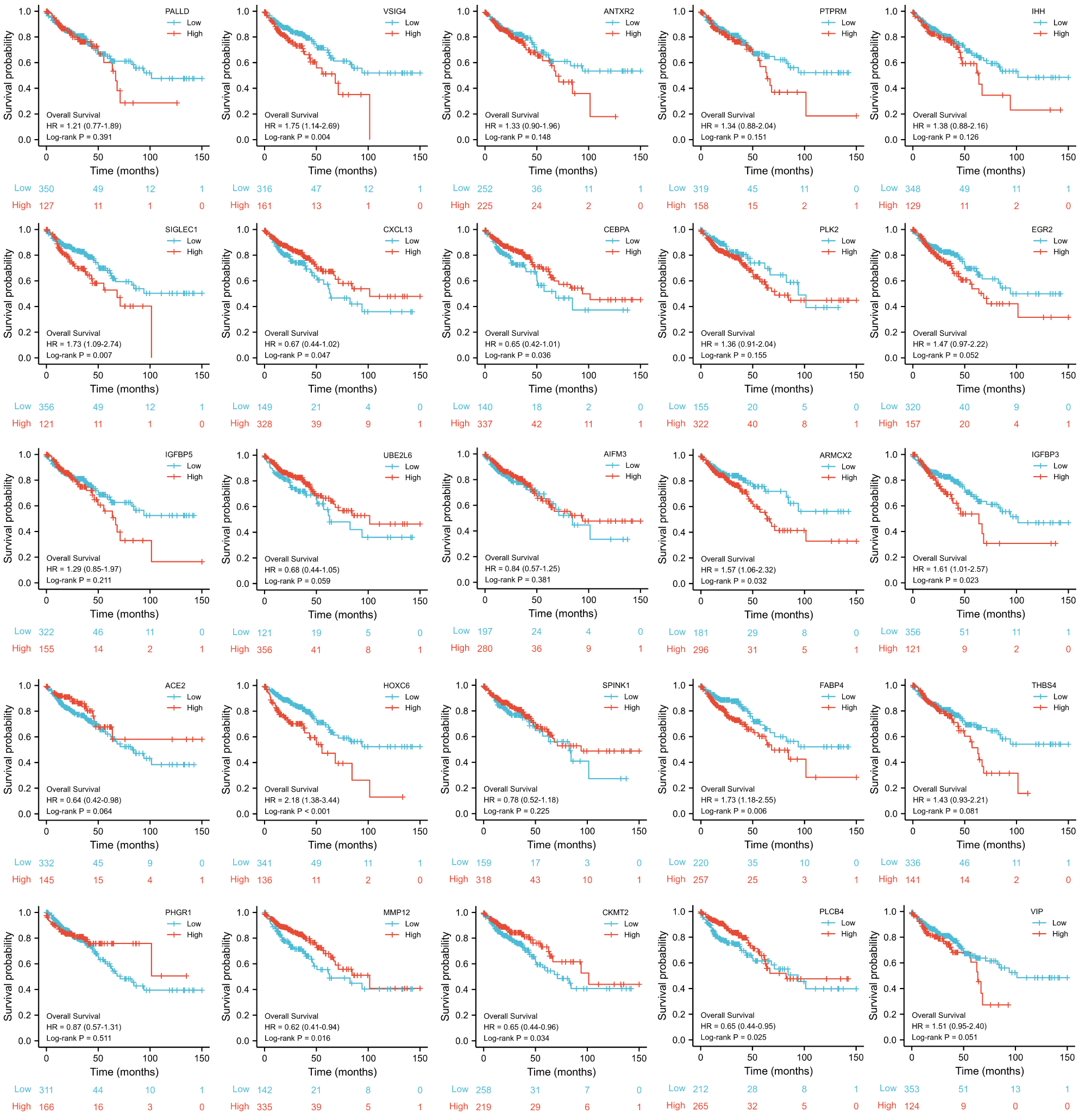


**Supplementary Figure 12.** Kaplan-Meier survival analysis of genes in NR-signature.

**Supplementary Table 1.** Clinicopathological characteristics of CRC patients in TCGA.

| Characteristic | levels | Overall |
| --- | --- | --- |
| n |  | 478 |
| Gender, n (%) | Female | 226 (47.3%) |
|  | Male | 252 (52.7%) |
| Age, n (%) | <=65 | 194 (40.6%) |
|  | >65 | 284 (59.4%) |
| Pathologic stage, n (%) | Stage I | 81 (16.9%) |
|  | Stage II | 187 (39.1%) |
|  | Stage III | 133 (27.8%) |
|  | Stage IV | 66 (13.8%) |
| T stage, n (%) | T1 | 11 (2.3%) |
|  | T2 | 83 (17.4%) |
|  | T3 | 323 (67.6%) |
|  | T4 | 60 (12.6%) |
| N stage, n (%) | N0 | 284 (59.4%) |
|  | N1 | 108 (22.6%) |
|  | N2 | 86 (18%) |
| M stage, n (%) | M0 | 349 (73.0%) |
|  | M1 | 66 (13.8%) |
| OS event, n (%) | Alive | 375 (78.5%) |
|  | Dead | 103 (21.5%) |

**Supplementary Table 2.** Clinicopathological characteristics of CRC patients in GSE39582.

| Characteristic | levels | Overall |
| --- | --- | --- |
| n |  | 566 |
| Gender, n (%) | Female | 256 (45.2%) |
|  | Male | 310 (54.7%) |
| Age, n (%) | <=65 | 222 (39.2%) |
|  | >65 | 343 (60.6%) |
| Pathologic stage, n (%) | Stage I | 33 (5.8%) |
|  | Stage II | 264 (46.6%) |
|  | Stage III | 205 (36.2%) |
|  | Stage IV | 60 (10.6%) |
| T stage, n (%) | T1 | 11 (1.9%) |
|  | T2 | 45 (7.9%) |
|  | T3 | 367 (64.8%) |
|  | T4 | 119 (21.0%) |
| N stage, n (%) | N0 | 302 (53.3%) |
|  | N1 | 134 (23.6%) |
|  | N2 | 98 (17.3%) |
|  | N3 | 6 (1.0%) |
| M stage, n (%) | M0 | 482 (85.1%) |
|  | M1 | 61 (10.7%) |
| OS event, n (%) | Alive | 371 (65.5%) |
|  | Dead | 191 (33.7%) |

**Supplementary Table 3.** Clinicopathological characteristics of CRC patients in GSE17538.

| Characteristic | levels | Overall |
| --- | --- | --- |
| n |  | 232 |
| Gender, n (%) | Female | 110 (47.4%) |
|  | Male | 122 (52.5%) |
| Age, n (%) | <=65 | 116 (50.0%) |
|  | >65 | 116 (50.0%) |
| Pathologic stage, n (%) | Stage I | 28 (12.0%) |
|  | Stage II | 72 (31.0%) |
|  | Stage III | 76 (32.7%) |
|  | Stage IV | 56 (24.1%) |
| OS event, n (%) | Alive | 139 (59.9%) |
|  | Dead | 93 (40.1%) |

**Supplementary Table 4.** Results of the univariate Cox analysis and Kaplan-Meier survival analysis in CRC patients based on the NRGs.

| Gene name | HR | HR.95L | HR.95H | 1. value   (Univariate COX analysis) | P-value  (KM survival analysis) |
| --- | --- | --- | --- | --- | --- |
| FADD | 1.132423096 | 0.860167703 | 1.49085122 | 0.375418695 | 0.088251732 |
| FAS | 1.002299261 | 0.888964799 | 1.130082776 | 0.970076302 | 0.123186659 |
| FASLG | 0.904938067 | 0.727040038 | 1.126365622 | 0.371089123 | 0.026655539 |
| MLKL | 1.01175838 | 0.787578631 | 1.29974961 | 0.927119184 | 0.31490358 |
| RIPK1 | 1.450421562 | 1.06577145 | 1.973896664 | 0.018024772 | 0.001430155 |
| RIPK3 | 0.729291573 | 0.567205466 | 0.937695826 | 0.013832405 | 0.000222411 |
| TLR3 | 0.927277998 | 0.796276449 | 1.079831617 | 0.3312513 | 0.038573211 |
| TNF | 0.924945933 | 0.730199031 | 1.171632586 | 0.517758317 | 0.019440898 |
| TSC1 | 1.352054381 | 1.040210536 | 1.757385632 | 0.024155154 | 0.004352265 |
| TRIM11 | 1.032147158 | 0.759075865 | 1.403453602 | 0.840063864 | 0.248160436 |
| CASP8 | 0.83123624 | 0.643084001 | 1.074437686 | 0.158055134 | 0.08126031 |
| ZBP1 | 0.812756309 | 0.674143104 | 0.979870318 | 0.029772034 | 0.003366154 |
| MAPK8 | 0.97445967 | 0.723571329 | 1.31234007 | 0.864740163 | 0.26273263 |
| IPMK | 1.035056961 | 0.841915234 | 1.272506862 | 0.743677679 | 0.105805197 |
| ITPK1 | 1.115112043 | 0.837179361 | 1.485314766 | 0.456319924 | 0.085012742 |
| SIRT3 | 0.979889834 | 0.662754495 | 1.448777932 | 0.918896136 | 0.005507622 |
| MYC | 0.793937012 | 0.695026631 | 0.906923491 | 0.000676076 | 0.000197299 |
| TNFRSF1A | 1.263967873 | 0.97314258 | 1.641706793 | 0.079105885 | 0.003107965 |
| TNFSF10 | 0.906738266 | 0.781185646 | 1.052469776 | 0.197936435 | 0.003786892 |
| TNFRSF1B | 0.797111051 | 0.668186516 | 0.950911179 | 0.011764095 | 0.003047697 |
| TRAF2 | 1.029754515 | 0.771355654 | 1.374715225 | 0.842342079 | 0.142667876 |
| PANX1 | 1.383814012 | 1.092646315 | 1.752571894 | 0.007037766 | 0.000420113 |
| OTULIN | 0.763074479 | 0.539654284 | 1.07899201 | 0.126059343 | 0.094614811 |
| CYLD | 1.247599032 | 0.968680609 | 1.606828226 | 0.086621425 | 0.016143965 |
| USP22 | 1.01443434 | 0.73844042 | 1.393581666 | 0.929515001 | 0.130492903 |
| MAP3K7 | 1.439170028 | 1.11957062 | 1.850004218 | 0.004490404 | 0.003211701 |
| SQSTM1 | 1.036797979 | 0.790917555 | 1.3591177 | 0.793593831 | 0.276414894 |
| STAT3 | 0.97271118 | 0.736511315 | 1.284660562 | 0.845430313 | 0.120197904 |
| DIABLO | 0.907450547 | 0.635146231 | 1.296499066 | 0.593687306 | 0.098643474 |
| DNMT1 | 0.944659758 | 0.772440376 | 1.155276298 | 0.579313393 | 0.032178744 |
| CFLAR | 1.026954052 | 0.780329491 | 1.351524757 | 0.849455638 | 0.209370877 |
| BRAF | 1.21878946 | 0.950344614 | 1.563062205 | 0.11905998 | 0.090382383 |
| AXL | 1.233505311 | 1.068130648 | 1.424484313 | 0.00427169 | 2.60E-05 |
| ID1 | 0.953791547 | 0.886487118 | 1.026205905 | 0.205111613 | 0.001831705 |
| CDKN2A | 1.208827234 | 1.075879825 | 1.358203072 | 0.001421228 | 0.000744375 |
| HSPA4 | 0.949182294 | 0.74055758 | 1.216579306 | 0.680447511 | 0.119101024 |
| BCL2 | 0.757077293 | 0.612523892 | 0.935744769 | 0.010044008 | 0.001477479 |
| STUB1 | 0.939202503 | 0.72374918 | 1.218794254 | 0.637090026 | 0.022049527 |
| FLT3 | 0.811079786 | 0.56024365 | 1.174222 | 0.267347947 | 0.005638452 |
| HAT1 | 0.910087938 | 0.750735124 | 1.103265357 | 0.337402193 | 0.040130838 |
| SIRT2 | 1.043272447 | 0.754986375 | 1.441638464 | 0.797392921 | 0.18632165 |
| SIRT1 | 1.19201955 | 0.966782464 | 1.469731465 | 0.10021416 | 0.011021424 |
| PLK1 | 0.74618561 | 0.628466798 | 0.885954462 | 0.000830931 | 9.44E-05 |
| MPG | 0.824824766 | 0.647930456 | 1.05001376 | 0.117887493 | 0.01044714 |
| BACH2 | 1.497297129 | 1.169074285 | 1.917670007 | 0.001387354 | 0.000600792 |
| GATA3 | 0.907772209 | 0.722707993 | 1.140225916 | 0.405499435 | 0.004757371 |
| MYCN | 0.828845774 | 0.698325064 | 0.983761506 | 0.031774889 | 0.014651863 |
| ALK | 0.998323135 | 0.624934379 | 1.594805974 | 0.99439724 | 0.139394052 |
| ATRX | 1.125836549 | 0.938531105 | 1.350523097 | 0.201723975 | 0.083266768 |
| TERT | 1.073805941 | 0.86607707 | 1.331358651 | 0.516222025 | 0.017748346 |
| SLC39A7 | 0.99186065 | 0.812393117 | 1.210974747 | 0.936037036 | 0.116745024 |
| SPATA2 | 0.762920503 | 0.629929659 | 0.923988395 | 0.005624746 | 0.003522338 |
| RNF31 | 0.977524634 | 0.717349035 | 1.332063422 | 0.885523174 | 0.190229724 |
| IDH1 | 0.765058944 | 0.594728789 | 0.984171607 | 0.037148222 | 0.026972331 |
| IDH2 | 0.7966551 | 0.672745644 | 0.943386783 | 0.008398098 | 0.001600244 |
| KLF9 | 1.078518821 | 0.917368779 | 1.267977367 | 0.359956295 | 0.008283596 |
| HDAC9 | 1.473255987 | 1.146267141 | 1.893523007 | 0.002477497 | 0.000173447 |
| HSP90AA1 | 1.106935212 | 0.890701577 | 1.375663404 | 0.359574075 | 0.02663265 |
| LEF1 | 1.235674794 | 1.066807602 | 1.431272324 | 0.004764535 | 0.003990256 |
| BNIP3 | 1.005014805 | 0.932839419 | 1.082774524 | 0.895334086 | 0.179565076 |
| CD40 | 0.997998873 | 0.862787913 | 1.154399286 | 0.978485383 | 0.035678669 |
| BCL2L11 | 1.268720072 | 0.972161873 | 1.655743416 | 0.079752446 | 0.010406697 |
| EGFR | 1.09714129 | 0.888785943 | 1.354340737 | 0.38826647 | 0.02509502 |
| DDX58 | 1.043387307 | 0.895376363 | 1.215865326 | 0.586345219 | 0.082852854 |
| TARDBP | 1.006213055 | 0.764186978 | 1.324891344 | 0.964806828 | 0.11808721 |
| APP | 1.042283476 | 0.823506536 | 1.31918181 | 0.73044975 | 0.278029408 |
| TNFRSF21 | 1.162278185 | 0.945209468 | 1.429197046 | 0.153944707 | 0.012954676 |

**Supplementary Table 5. Primer sequences used in the study.**

| Primer name | Primer sequences |
| --- | --- |
| Primers for real-time PCR: |  |
| *ARMCX2* sense: | 5’-ACACTGGGGCTATACCGAAAG-3’ |
| *ARMCX2* antisense: | 5’-GACCTGGTTACACCCTTGCC-3’ |
| *VSIG4* sense: | 5’-GGGGCACCTAACAGTGGAC-3’ |
| *VSIG4* antisense: | 5’-GTCTGAGCCACGTTGTACCAG-3’ |
| *IGFBP3* sense: | 5’-AGAGCACAGATACCCAGAACT-3’ |
| *IGFBP3* antisense: | 5’-GGTGATTCAGTGTGTCTTCCATT-3’ |
| *FABP4* sense: | 5’-ACTGGGCCAGGAATTTGACG-3’ |
| *FABP4* antisense: | 5’-CTCGTGGAAGTGACGCCTT-3’ |
| *MMP12* sense: | 5’-GATCCAAAGGCCGTAATGTTCC-3’ |
| *MMP12* antisense: | 5’-TGAATGCCACGTATGTCATCAG-3’ |
| *SIGLEC1* sense: | 5’-AGGCGTACCCCATCCTTGA-3’ |
| *SIGLEC1* antisense: | 5’-GCTTGAGGTGTAGATGTGTCC-3’ |
| *CXCL13* sense | 5’-GCTTGAGGTGTAGATGTGTCC-3’ |
| *CXCL13* antisense | 5’-CCCACGGGGCAAGATTTGAA-3’ |
| *CKMT2* sense: | 5’-AGGTGACACCCAACGGCTA-3’ |
| *CKMT2* antisense: | 5’-TGACGGGGTCAAAAAGGTCAG-3’ |
| *CEBPA* sense: | 5’-TATAGGCTGGGCTTCCCCTT-3’ |
| *CEBPA* antisense: | 5’-AGCTTTCTGGTGTGACTCGG-3’ |
| *HOXC6* sense: | 5’-GACCGACCAGGTAAAGGCAA-3’ |
| *HOXC6* antisense: | 5’-TCTTCTAGGGAAGCCGGTCA-3’ |
